# Structural and Functional Impacts of SARS-CoV-2 Spike Protein Mutations: Insights From Predictive Modeling and Analytics

**DOI:** 10.2196/73637

**Published:** 2025-12-08

**Authors:** Edem K Netsey, Samuel M Naandam, Joseph Asante Jnr, Kuukua E Abraham, Aayire C Yadem, Gabriel Owusu, Jeffrey G Shaffer, Sudesh K Srivastav, Seydou Doumbia, Ellis Owusu-Dabo, Chris E Morkle, Desmond Yemeh, Stephen Manortey, Ernest Yankson, Mamadou Sangare, Samuel Kakraba

**Affiliations:** 1 Department of Mathematics and Information Communication Technology, Dambai College of Education, Dambai, Ghana; 2 Department of Mathematics, School of Physical Sciences, University of Cape Coast, Cape Coast, Ghana; 3 Department of Geriatrics, School of Medicine, University of Arkansas for Medical Sciences, Little Rock, AR, United States; 4 Department of Mathematics, Memphis Shelby County Schools, Memphis, TN, United States; 5 Research and Development, CytoAstra LLC, Little Rock, AR, United States; 6 Office of Research, Celia Scott Weatherhead School of Public Health and Tropical Medicine at Tulane University, New Orleans, LA, United States; 7 Department of Biostatistics and Data Science, Celia Scott Weatherhead School of Public Health and Tropical Medicine at Tulane University, 1440 Canal Street, New Orleans, LA, 70112, United States, 1 5049882475; 8 Department of Public Health, Faculty of Medicine, Malaria Research and Training Center, University of Sciences, Techniques and Technologies of Bamako, Bamako, Mali; 9 School of Public Health, Kwame Nkrumah University of Science and Technology, Kumasi, Ghana; 10 Department of Mathematics, Suhum Senior High School, Suhum, Ghana; 11 Tulane Center for Aging, School of Medicine, Tulane University, New Orleans, LA, United States; 12 Department of Community Health, Ensign Global University, Kpong, Ghana; 13 Laboratory of Malaria and Vector Research (LMVR), National Institute of Allergy and Infectious Diseases (NIAID), Rockville, MD, United States

**Keywords:** COVID-19, graph-theoretic modeling, 6M0J, predictive modeling, unsupervised machine learning, molecular dynamics simulations, E484A, SARS-CoV-2 spike mutations (N501Y, E484A, L452R, N440K, K417N)

## Abstract

**Background:**

The COVID-19 pandemic requires a deep understanding of SARS-CoV-2, particularly how mutations in the spike receptor-binding domain (RBD) chain E affect its structure and function. Current methods lack comprehensive analysis of these mutations at different structural levels.

**Objective:**

This study aims to analyze the impact of specific COVID-19–associated point mutations (N501Y, L452R, N440K, K417N, and E484A) on the SARS-CoV-2 spike RBD structure and function using predictive modeling, including a graph-theoretic model, protein modeling techniques, and molecular dynamics simulations.

**Methods:**

The study used a multitiered graph-theoretic framework to represent protein structure across 3 interconnected levels. This model incorporated 19 top-level vertices, connected to intermediate graphs based on 6-angstrom proximity within the protein’s 3D structure. Graph-theoretic molecular descriptors or invariants were applied to weigh vertices and edges at all levels. The study also used Iterative Threading Assembly Refinement (I-TASSER) to model mutated sequences and molecular dynamics simulation tools to evaluate changes in protein folding and stability compared to the wildtype.

**Results:**

A total of 3 distinct predictive modeling and analytical approaches successfully identified structural and functional changes in the SARS-CoV-2 spike RBD (chain E) resulting from point mutations. The novel graph-theoretic model detected notable structural changes, with N501Y and L452R showing the most pronounced effects on conformation and stability compared to the wildtype. K147N and E484A mutations demonstrated less significant impacts compared to the severe mutations, N501Y and L452R. Ab initio modeling and molecular simulation dynamics findings corroborated the results from graph-theoretic analysis. The multilevel analytical approach provided a comprehensive visualization of mutation effects, deepening our understanding of their functional consequences.

**Conclusions:**

This study advanced our understanding of SARS-CoV-2 spike RBD mutations and their implications. The multifaceted approach characterized the effects of various mutations, identifying N501Y and L452R as having the most substantial impact on RBD conformation and stability. The findings have important implications for vaccine development, therapeutic design, and variant monitoring. Our research underscores the power of combining multiple predictive analytical approaches in virology, contributing valuable knowledge to ongoing efforts against the COVID-19 pandemic and providing a framework for future studies on viral mutations and their impacts on protein structure and function.

## Introduction

### Background

The COVID-19 pandemic, caused by SARS-CoV-2, had a devastating global impact, with hundreds of millions of confirmed cases and millions of deaths worldwide [1-4]. The virus’ spike protein shares significant structural and sequence similarities with the severe acute respiratory syndrome virus from 2003, including the use of the angiotensin-converting enzyme 2 (ACE2) receptor for cell entry. The SARS-CoV-2 spike protein’s receptor-binding domain (RBD) has undergone various mutations, each with distinct impacts on viral behavior and infectivity [5]. The wildtype phenotype serves as a baseline, exhibiting no significant changes or enhanced impacts [1]. Protein folding, maintenance, mutation, aggregation, neurodegenerative diseases, and COVID-19 are interconnected through complex biological processes [6-8]. Except for intrinsically disordered proteins, most proteins must fold correctly to function properly, but mutations can disrupt this process, leading to misfolding and aggregation. Cells have protective mechanisms to maintain protein homeostasis, but when these fail, aggregates can form and contribute to neurodegenerative diseases like Alzheimer disease (AD) and Parkinson disease [6-10]. COVID-19 has been linked to AD, Parkinson disease, and other neurodegenerative diseases through various mechanisms [11-13]. The SARS-CoV-2 spike protein contains aggregation-prone regions that can form amyloid aggregates, potentially contributing to neurological complications [14-16]. In addition, the virus’ 3CL protease has been shown to induce tau protein aggregation, a hallmark of neurodegenerative diseases. Genetic studies have revealed a causal association between COVID-19 hospitalization and increased risk of AD [17]. The virus may accelerate neurodegeneration through inflammation, microvascular injury, and prion-like spread of misfolded protein. These aggregates were observed in vitro using various experimental techniques, including fluorescence spectroscopy and electron microscopy [18-20].

Specific mutations have emerged that alter the virus’ characteristics in different ways. Mutations such as K417N, associated with the Beta, Gamma, and Omicron variants, demonstrate moderate severity due to their role in immune evasion [21-23]. These mutations reduce antibody neutralization, potentially compromising the effectiveness of vaccines and natural immunity [24]. Similarly, N440K and S477N mutations, linked to localized outbreaks, show mild impacts by enhancing binding to the ACE2 receptor, which increases infectivity without causing major antigenic alterations [25,26]. More concerning are mutations like T478K and L452R, found in Delta and Omicron variants, which display moderate to severe consequences. L452R, in particular, is associated with immune evasion and increased transmission due to its resistance to neutralizing antibodies [27]. The E484 mutations (A, K, Q), present in Beta, Gamma, and other variants, exhibit moderate to severe immune escape capabilities, significantly reducing neutralization by vaccine-induced or convalescent sera [28]. Of note is the profound N501Y mutation, observed in Alpha, Beta, and Omicron variants. This mutation is classified as very severe due to its dual role in enhancing binding affinity to the ACE2 receptor and aiding in immune escape [29]. N501Y has been a pivotal factor in increasing transmissibility and contributing to widespread outbreaks [30]. These diverse mutations in the SARS-CoV-2 RBD demonstrate varying degrees of severity and impact, ranging from mild infectivity increases to severe immune evasion and transmissibility. The evolving nature of these mutations underscores the critical importance of continuous genomic surveillance and adaptive vaccine strategies in combating the COVID-19 pandemic [31-33]. As SARS-CoV-2 has spread, it has evolved into various strains, with the D614G mutation becoming nearly ubiquitous. Several “variants of concern” (VOCs) have emerged, characterized by mutations that may affect viral behavior and immune evasion [34-38]. These VOCs, identified in different regions, include B.1.1.7 (UK), P.1 and P.2 (Brazil), B.1.351 (South Africa), B.1.617 (India), and B.1.526 (US), among others [39]. The emergence of these variants has significant implications for treatment strategies and vaccine efficacy. Some variants, such as B.1.351, have shown resistance to neutralization by convalescent plasma and certain monoclonal antibody treatments [40]. In addition, in vitro studies suggest that sera from vaccinated individuals may have reduced neutralizing capacity against variants with specific mutations, like E484K and N501Y [23,41,42]. While VOCs are defined by specific mutation patterns, the interplay between these mutations in affecting viral behavior is not fully understood [43-46]. Analysis of a large dataset of SARS-CoV-2 spike sequences revealed hundreds of amino acid variants, with a significant number occurring in the RBD [47,48]. Machine learning algorithms have revolutionized data analysis by uncovering hidden patterns in datasets and addressing critical questions across various disciplines, including disease diagnostics, among others [49-52]. Unsupervised learning techniques like clustering and dimensionality reduction reveal intrinsic structures in data [53], enabling applications such as customer segmentation, anomaly detection, and in the field of bioinformatics [54]. To better understand the emergence and spread of new variants, novel bioinformatics approaches are being developed to identify spatially and temporally correlated mutations [55,56]. The evolving nature of SARS-CoV-2 suggests that future vaccine design may need to be tailored to address the specific strain ensembles prevalent in different regions [57-59]. This approach could enhance vaccine efficacy against locally dominant variants and potentially provide broader protection against emerging strains.

### Previous Work

Recent advancements in graph-theoretic modeling have provided valuable insights into the complex relationship between point mutations and disease phenotypes [60,61]. Researchers have developed various graph-based approaches to model and analyze the intricate connections between genes, mutations, and phenotypes. These methods leverage the ability of graphs to represent complex biological relationships and interactions [62-75]. This approach has been particularly effective in elucidating the structural and functional impacts of genetic variations in proteins associated with hereditary disorders. One application of graph theory in mutation analysis involves the use of protein-protein interaction networks to identify discriminative subnetworks associated with specific diseases [9,76-84], allowing researchers to uncover patterns of mutations that may collectively contribute to phenotypes, going beyond the limitations of single-gene analyses.

Additionally, other studies exemplify the power of this methodology in understanding the molecular basis of point mutation-associated diseases like cystic fibrosis and sickle cell disease (SCD). Previously, we developed a hierarchical graph-theoretic model to investigate the effects of point mutations on the NBD2 domain of the CFTR protein, which is implicated in cystic fibrosis [60,85]. By constructing a multilevel graph (nested graph) representation of interacting amino acid residues, they developed a nested graph capable of quantifying both local and global structural changes resulting from virtual point mutations. This innovative method enabled the differentiation of mild mutations (such as Y1219G and G1271E) from severe ones (like N1303K) when compared to the wildtype, demonstrating the sensitivity and relevance of their graph-theoretic methods and resulting molecular descriptors (graph invariants) in analyzing complex biological networks. Building on this framework, Netsey et al [61] applied similar graph-theoretic techniques to explore the impact of point mutations on the hemoglobin protein (1A3N) in SCD. Using author-adopted and molecular descriptors from our previous works [60,85], the authors successfully captured the structural effects of various mutations, including E6V, V23I, and K82N associated with SCD. Their analysis not only distinguished mild mutations from the wildtype but also highlighted the significant devastation caused by the severe E6V mutation, further validating the usability of graph-theoretic molecular nested graphs in understanding many disease mechanisms. These studies collectively demonstrate the power of graph-theoretic modeling in bridging the gap between genetic mutations and their phenotypic manifestations in complex diseases. In recent years, the fields of ab initio modeling and molecular dynamics (MD) simulations have revolutionized our approach to studying intricate biological systems and networks [86-89]. These advanced computational methods enable researchers to create detailed models of biological entities, ranging from individual proteins to entire cellular structures, with unprecedented atomic-level precision [90]. By combining these techniques with systems biology principles, researchers can now explore the complex relationships between genetic alterations and disease manifestations. These sophisticated MD simulations provide a unique window into the molecular consequences of mutations, allowing scientists to track cascading effects from the smallest cellular components up to organism-wide changes [86-89]. This multitiered modeling strategy offers valuable insights into disease mechanisms, illuminating how specific genetic changes can influence protein behavior, disrupt cellular functions, and ultimately result in diverse clinical phenotypes. The fusion of structural data with network analysis significantly enhances our ability to predict and understand the connections between genetic makeup and disease outcomes. This integrated approach provides a more holistic view of the intricate relationships between genotypes and phenotypes in complex disorders, paving the way for more targeted and effective therapeutic interventions [91]. By providing a computational framework for quantifying structural changes at multiple levels, this approach offers a promising avenue for predicting disease severity and potentially informing therapeutic strategies.

### Goal of This Study

In this study, we hypothesized that specific point mutations in the SARS-CoV-2 spike protein significantly alter its structural conformation, dynamic behavior, and stability, thereby influencing its binding affinity, immune escape potential, and overall viral fitness. To test this hypothesis, we employed a multifaceted computational approach combining graph-theoretic modeling with ab initio protein structure prediction and MD simulations to create a comprehensive analysis framework. Using the Iterative Threading Assembly Refinement (I-TASSER) [92] platform, we generated detailed 3D models of mutated spike proteins, which allowed us to visualize how point mutations affect protein structure, including potential changes in binding sites and overall conformation. We then conducted MD simulations to study the dynamic behavior of these mutated proteins, revealing how mutations impact protein movement and stability compared to the wildtype. By integrating these computational methods with our graph-based approach, we developed a holistic understanding of mutation effects, bridging the gap between genetic changes and their structural consequences. This comprehensive approach provided insights into both sequence-level alterations and their macromolecular impacts, enhancing our understanding of SARS-CoV-2’s evolution and potential behavioral changes. Through the use of these complementary methods, we were able to construct a more complete picture of how spike protein mutations influence the virus’s structure and function.

## Methods

### Graph-Theoretic Model of SARS-CoV-2 Spike RBD (Chain E)

This work extends our previous graph-theoretic modeling approaches [60,85] by focusing specifically on the chain E of the SARS-CoV-2 spike protein and expanding combinatorial descriptors to include edge-weight assignments, among others. In this study, we focused exclusively on the spike protein, and human ACE2 (hACE2) was not included as part of the analysis. The study incorporates molecular indices from earlier research [60,85] and introduces additional graph invariants. As a result, the molecular descriptors for subdomain graphs, the chain E of the SARS-CoV-2 spike protein, and the examined mutations are tailored to chain E of the SARS-CoV-2 spike RBD protein.

### Subsequence Partition of SARS-CoV-2 Spike Protein Chain E

**Figure 1.**
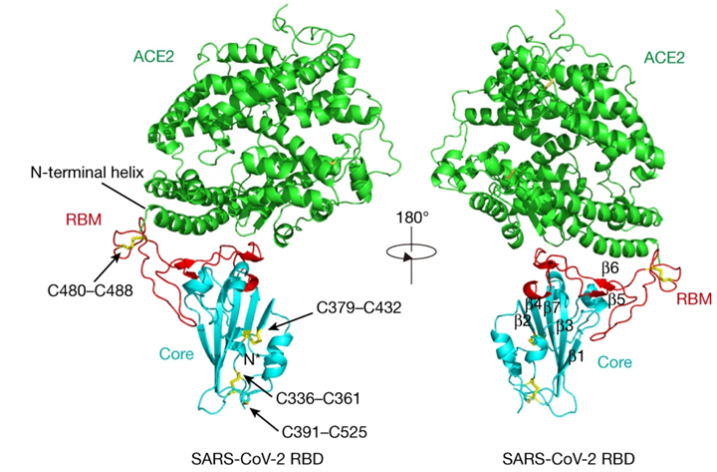
Overall structure of the SARS-CoV-2 receptor-binding domain (RBD) in complex with human angiotensin-converting enzyme 2 (ACE2). The crystal structure of the SARS-CoV-2 spike protein RBD bound to human ACE2 receptor is shown in two orientations related by a 180° rotation. ACE2 (green) forms the primary attachment point for viral entry, with its N-terminal helix directly accommodating the concave surface of the viral RBD. The SARS-CoV-2 RBD consists of 2 major regions: the core structure (cyan) containing a twisted 5-stranded antiparallel β sheet (β1-β7, labeled in right panel), and the receptor-binding motif (RBM; red) that directly interfaces with ACE2. A total of 4 critical disulfide bonds are highlighted as yellow sticks and indicated by arrows: 3 within the core (C336–C361, C379–C432, and C391–C525) stabilizing the β sheet structure, and one (C480–C488) connecting loops at the distal end of the RBM. The N-terminal helix of ACE2, which serves as the primary interaction surface with the viral RBD, is specifically labeled. This binding interface is the initial critical point of contact during viral infection and serves as a major determinant of host range and transmissibility. The structure was determined by x-ray crystallography at 2.45 Å resolution (Protein Data Bank [PDB] 6M0J). This figure was adapted from Lan et al [93].

Figure 1 shows the tertiary crystal structure of SARS-CoV-2 spike RBD bound with ACE2 [93], which was retrieved from the Protein Data Bank [94] (PDB; 6M0J) and visualized using UCSF Chimera [95].

**Figure 2.**
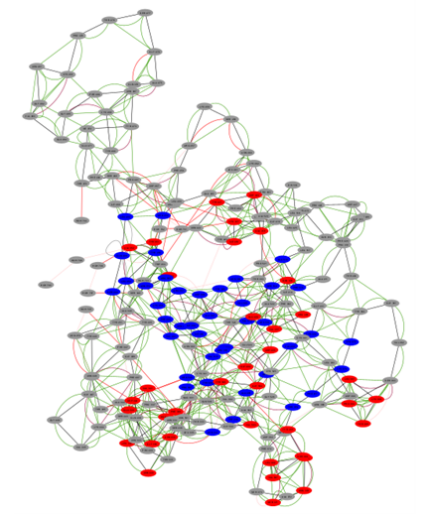
Network graph of SARS-CoV-2 spike receptor-binding domain (RBD). This figure illustrates a network graph representation of the SARS-CoV-2 spike protein's RBD, derived from the crystal structure 6M0J obtained from the Protein Data Bank. Each vertex in the graph represents an individual amino acid residue of the RBD. Edges connecting these vertices were established using a 6-angstrom proximity threshold, where two residues are connected if any of their atoms are within 6Å of each other. This representation provides a comprehensive view of the RBD structure, from individual amino acids to their network interactions.

To provide a more comprehensive understanding of the SARS-CoV-2 Spike RBD structure, we generated a network graph for only the SARS-CoV-2 spike protein, without the ACE2, using Cytoscape (Cytoscape Consortium) [96]. Figure 2 illustrates this network visualization, offering valuable insights into the RBD’s intricate architecture and its potential functional significance. This graphical representation elucidates the complex relationships between various structural elements within the RBD, enhancing our understanding of its overall organization and possible mechanistic implications.

The spike protein RBD, corresponding to chain E of the structure [93], was meticulously divided into 19 subsequences (G1 to G19), while preserving the integrity of crucial biological information within the protein’s secondary structures. This partitioning approach, following our previously published method [60,85], enables a more detailed analysis of the RBD’s structure, which in turn informs the graph-theoretic modeling of the subsequence. During the partitioning for analyzing the SARS-CoV-2 RBD (chain E), we followed 3 key principles to maintain structural integrity and facilitate detailed analysis. We preserved binding sites and secondary structures, isolated different structural elements into separate subsequences, and limited each subsequence to 13 amino acid residues. To account for protein complexity, loop regions were given more flexibility, allowing for the inclusion of turns, 3/10-helices, and short alpha helices. This careful approach provided a comprehensive view of the RBD’s structure, enabling more in-depth analysis and modeling. The core structure of the SARS-CoV-2 RBD consists of a twisted 5-stranded antiparallel β sheet (β1, β2, β3, β4, and β7), interconnected by short helices and loops. This detailed structural breakdown sets the foundation for further study of the RBD’s composition and function [93,97]. Table 1 provides a comprehensive overview of the subsequence partition, including subsequence identifiers (G1 to G19), corresponding amino acid residues, secondary structure classification, corresponding subdomains, and reason for the partitions. This detailed structural breakdown facilitates in-depth analysis of the SARS-CoV-2 spike RBD (chain E).

**Table 1.** Subsequence partition of SARS-CoV-2 spike receptor-binding domain (chain E).

Subdomain graph	Subsequence	Amino acid sequence	Structural or functional regions
G1	TNLCP	333‐337	Coil and turn
G2	FGEVFNA	338‐344	Alpha helix, turn, genome variant site (339), and mutagenesis (343)
G3	TRFASVYA	345‐352	Alpha helix, coil, and genome variant site (346)
G4	WNRKRISNCV	353‐362	Beta sheet and coil
G5	ADYSVLYNSAS	363‐373	Alpha helix, coil, and genome variant site (371, 373)
G6	FSTFKCYG	374‐381	Beta sheet, coil, and genome variant site (375)
G7	VSPTKLNDLCF	382‐392	Coil and alpha helix
G8	TNVYADSFVIR	393‐403	Beta sheet and coil
G9	GDEVRQIAPG	404‐413	Alpha helix and coil
G10	QTGKIADYNYKLPDD	414‐428	Alpha helix, coil, and genome variant site (417)
G11	FTGCVIAWNS	429‐438	Beta sheet and coil
G12	NNLDSKVGGNY	439‐449	Alpha helix, coil, turn, and genome variant site (440, 446)
G13	NYLYRLFRK	450‐458	Beta sheet, coil, genome variant site (452, 453), and mutagenesis (452, 453)
G14	SNLKPFERDISTEIY	459‐473	Coil and turns
G15	QAGSTPCNGVEGFN	474‐487	Coil, turns, genome variant site (477, 478, 484), and mutagenesis (475, 483)
G16	CYFPLQSYGF	488‐497	Beta sheet, coil, genome variant site (490, 493, 496), and mutagenesis (490, 493)
G17	QPTNGVGYQ	498‐506	Alpha helix, coil, genome variant site (501, 505), and mutagenesis (501)
G18	PYRVVVLSFELLHA	507‐520	Beta sheet, coil, and mutagenesis (519)
G19	PATVCG	521‐526	Coil

### Subdomain Graphs (Corresponding to Subsequence Partitions) of SARS-CoV-2 Spike Protein Chain E

**Figure 3.**
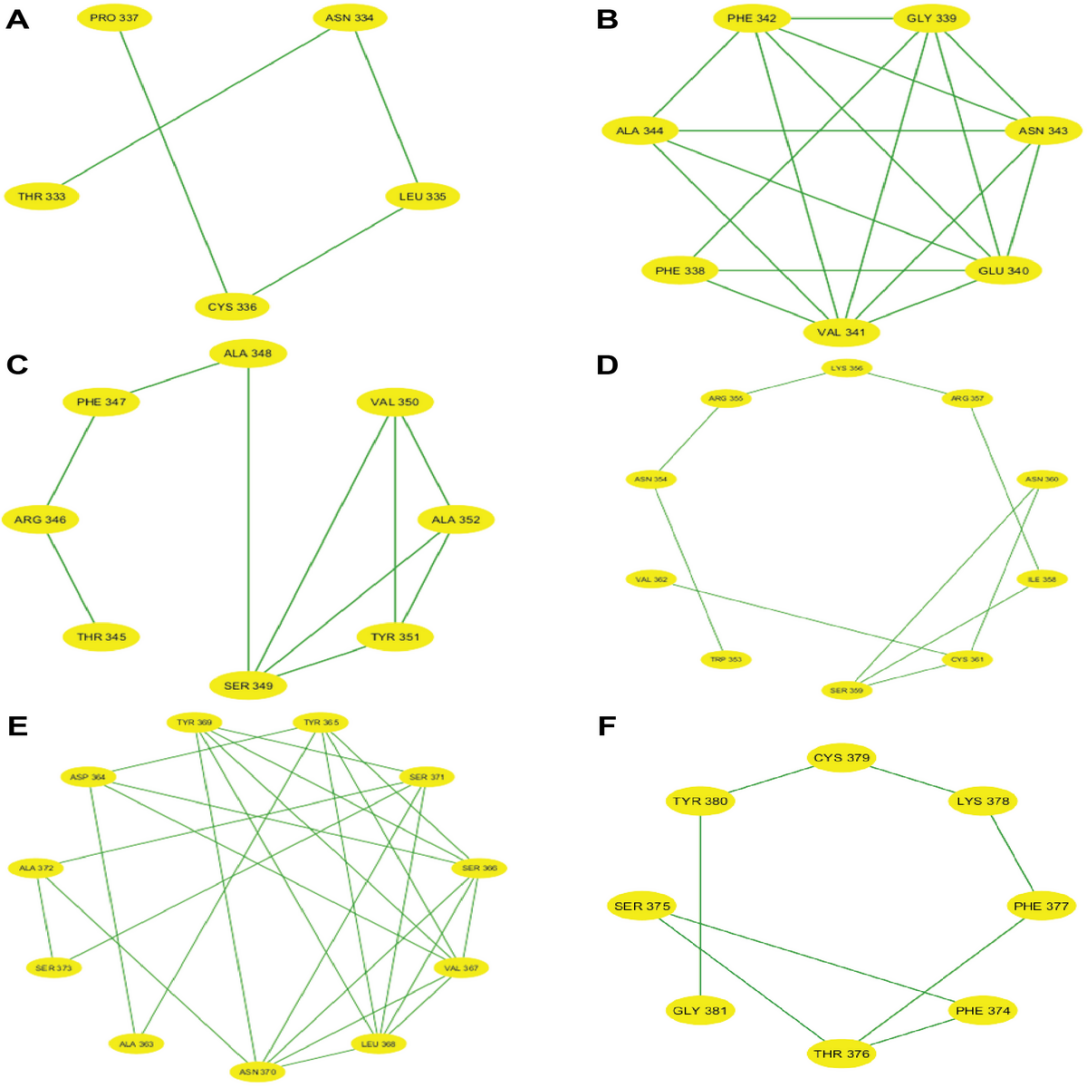
Nested interaction graphs (G1-G6) derived from ab initio modeling of receptor-binding domain subsequences using Iterative Threading Assembly Refinement (I-TASSER). Graphs represent local structural context of amino acids, with nodes as residues and edges indicating spatial proximity (≤6Å between residue centers of mass). (A) Subdomain graph G1 corresponding to subsequence S1. (B) Subdomain graph G2 corresponding to subsequence S2. (C) Subdomain graph G3 corresponding to subsequence S3. (D) Subdomain graph G4 corresponding to subsequence S4. (E) Subdomain graph G5 corresponding to subsequence S5. (F) Subdomain graph G6 corresponding to subsequence S6.

**Figure 4.**
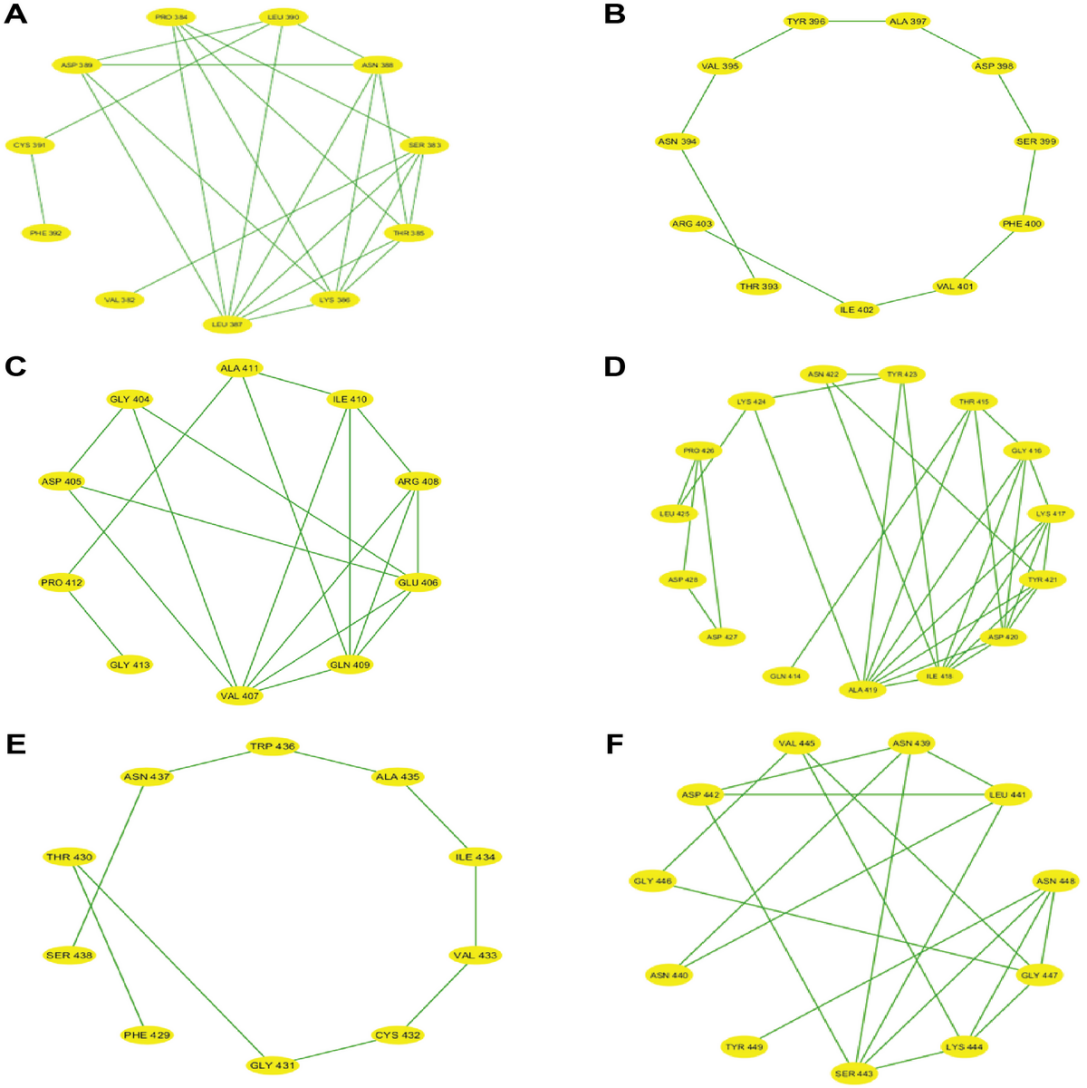
Nested interaction graphs (G7-G12) derived from ab initio modeling of receptor-binding domain subsequences using Iterative Threading Assembly Refinement (I-TASSER). Graphs represent local structural context of amino acids, with nodes as residues and edges indicating spatial proximity (≤6Å between residue centers of mass). (A) Subdomain graph G7 corresponding to subsequence S7. (B) Subdomain graph G8 corresponding to subsequence S8. (C) Subdomain graph G9 corresponding to subsequence S9. (D) Subdomain graph G10 corresponding to subsequence S10. (E) Subdomain graph G11 corresponding to subsequence S11. (F) Subdomain graph G12 corresponding to subsequence S12.

**Figure 5.**
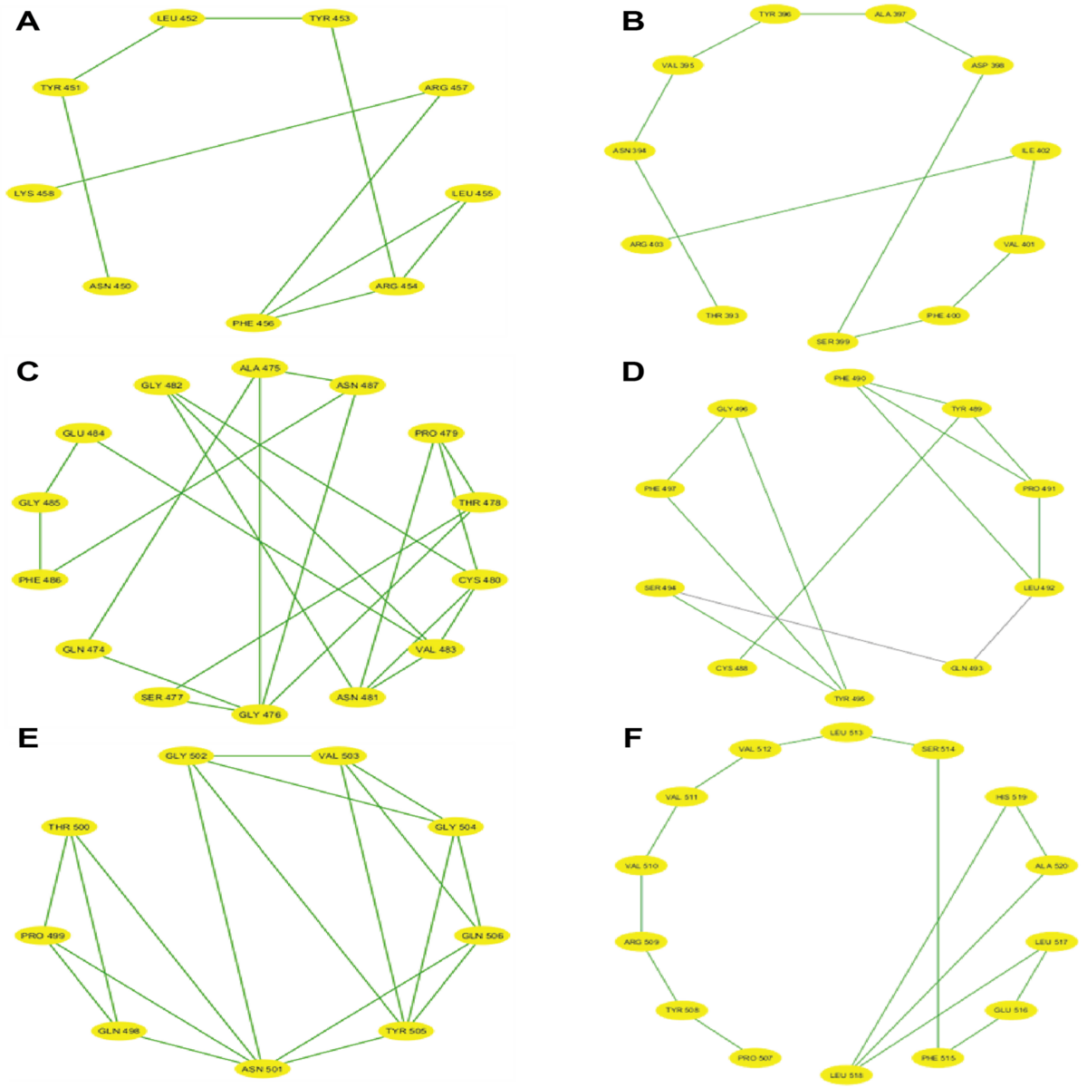
Nested interaction graphs (G13-G18) derived from ab initio modeling of receptor-binding domain subsequences using Iterative Threading Assembly Refinement (I-TASSER). Graphs represent local structural context of amino acids, with nodes as residues and edges indicating spatial proximity (≤6Å between residue centers of mass). (A) Subdomain graph G13 corresponding to subsequence S13. (B) Subdomain graph G14 corresponding to subsequence S14. (C) Subdomain graph G15 corresponding to subsequence S15. (D) Subdomain graph G16 corresponding to subsequence S16. (E) Subdomain graph G17 corresponding to subsequence S17. (F) Subdomain graph G18 corresponding to subsequence S18.

**Figure 6.**
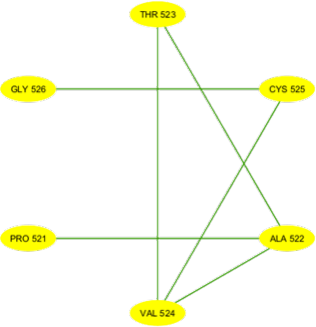
Nested interaction graphs (G19) derived from ab initio modeling of receptor-binding domain subsequences using Iterative Threading Assembly Refinement (I-TASSER). Graphs represent local structural context of amino acids, with nodes as residues and edges indicating spatial proximity (≤6Å between residue centers of mass). Subdomain graph G19 corresponding to subsequence S19.

After partitioning into subsequences, as shown in Table 1, we applied a sophisticated multistep graph-theoretic modeling approach. Using I-TASSER [92,98], a state-of-the-art protein structure prediction tool, we generated ab initio models for each subsequence, using a proximity threshold of 6 angstroms and determining end points based on each amino acid residue’s center of mass. These structural predictions were visualized using Cytoscape [96], a powerful network visualization software, resulting in 19 comprehensive subdomain graphs depicted in Figures 3-6 for all subdomain graphs. Each subdomain graph, corresponding to a previously classified subsequence in Table 1, offers a detailed visual representation of the structural and interaction patterns within each RBD subdomain.

**Figure 7.**
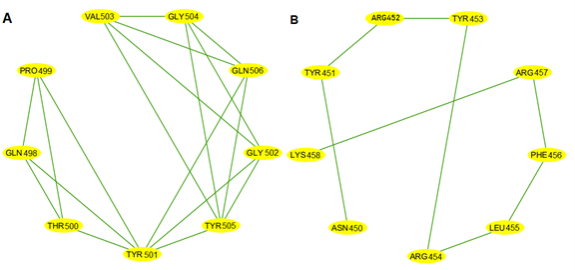
Subdomain graphs of SARS-CoV-2 spike protein receptor-binding domain (RBD; chain E) where N501Y and L452R mutations occur. (A) Subdomain graph for G13 containing N501Y mutation. (B) Subdomain graph for G17 containing L452R mutation. Ab initio models were generated for each subsequence, employing a proximity threshold of 6 angstroms and determining end points based on each amino acid residue's center of mass. These graphs illustrate the local structural context surrounding the mutation sites, with nodes representing amino acid residues and edges indicating spatial proximity. The central nodes (N501Y in A and L452R in B) highlight the locations of the severe mutations, while surrounding nodes depict neighboring residues that may influence or be affected by these mutations. This representation aids in visualizing potential structural and functional impacts of these critical mutations on the spike protein's RBD.

Figure 7 illustrates the subdomain graphs for subsequences G13 and G17 of the SARS-CoV-2 spike protein’s RBD in chain E. These specific subdomains are of particular interest, as they contain the locations of severe mutations N501Y and L452R, respectively. The graphical representation provides a detailed view of the structural context surrounding these critical mutation sites, offering insights into how these changes might affect the protein’s function and interactions.

This methodical approach not only enables a more nuanced understanding of the RBD’s structure but also lays the groundwork for further analysis of how mutations might affect these interactions and, consequently, the entire protein.

### Graph-Theoretic Model of SARS-CoV-2 Spike RBD (Chain E)

**Figure 8.**
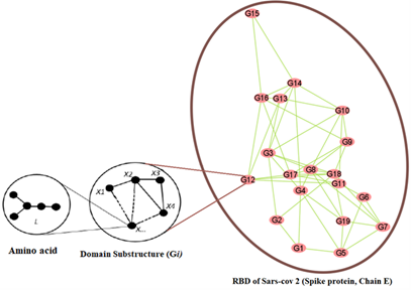
Hierarchical graph model of SARS-CoV-2 spike receptor-binding domain (RBD) chain E. This figure depicts a three-level hierarchical graph model of the SARS-CoV-2 spike RBD (chain E), illustrating the protein's structure and interactions at different scales. The lower level shows 20 vertex-weighted amino acids, the middle level presents 19 vertex-weighted subdomain graphs, and the top level displays a condensed view with single weighted vertices representing each subdomain. Edges between vertices are established using a 6-angstrom proximity threshold, providing a comprehensive view from individual amino acids to subdomain interactions.

Next, we modeled SARS-CoV-2 spike RBD corresponding to chain E of the structure protein to consist of 3 distinct levels, with each level offering a unique perspective on the protein’s structure and interactions. At the foundation, we have the lowest level, comprising 20 vertex-weighted amino acids representing the essential building blocks of the protein. Moving up, the middle level features 19 distinct vertex-weighted subgraphs (subdomain graphs), each corresponding to a specific subsequence of SARS-CoV-2 spike RBD corresponding to chain E (Table 1 and Figures 3-7) of the structure protein. In these subgraphs or subdomain graphs, individual amino acids are represented as vertices, with weights assigned based on graph invariants derived from the lower-level graphs. The top level of our model presents a more condensed view, where each subdomain graph from the middle level is consolidated into a single weighted vertex. The weights of these vertices are determined by molecular descriptors calculated from their respective subdomain graphs. To establish connections between vertices in the SARS-CoV-2 RBD corresponding to chain E nested domain graph, we used a proximity threshold of 6 angstroms between adjacent residues. A 6-angstrom proximity threshold is often used to define connections in the SARS-CoV-2 spike RBD graph because it captures meaningful atomic interactions, such as hydrogen bonds and van der Waals forces, essential for protein structure and function. This threshold ensures accurate representation of residue connectivity, aiding in understanding how the RBD stabilizes its structure and interacts with the ACE2 receptor [99]. In addition, it accommodates the dynamic flexibility of the RBD, reflecting both stable and transient interactions critical for receptor binding and regulation [100]. This balance of specificity and inclusivity makes it effective for modeling structural and functional relationships. Figure 8 provides a visual representation of our graph-theoretic model for the SARS-CoV-2 spike RBD corresponding to chain E, illustrating the hierarchical structure and interactions captured by this approach.

Building on our previous work [60,85], we used Cytoscape [96] to analyze the structural properties of subdomain graphs (referred to as mid-level graphs) by calculating a specific parameter: the change in molar mass between adjacent vertices normalized by the average degree of these graphs. This parameter, termed Δ*Md*, quantifies the variation in molar mass between two connected vertices, *R_i_* and *R_j_*, in the mid-level graphs, adjusted by the average connectivity (degree) of the graph. The results of this analysis are presented in Table 2 and are derived using equation 1, as established in our previous work [101]. Specifically, ΔMd represents the change in molar mass per average degree along the edge connecting vertices *R_i_* and *R_j_*. It is calculated using the following equation:


(1) $$\Delta Md=\left|\frac{R_{i}-R_{j}}{\bar{d}}\right|g/deg$$

**Table 2.** Molar mass as weighted degrees of subdomain.

Subdomain	Molar mass (g/mol)
G1	618.7
G2	890.9
G3	1040.1
G4	1437.6
G5	1369.4
G6	1078.3
G7	1416.6
G8	1464.5
G9	1203.3
G10	1993.2
G11	1259.4
G12	1360.4
G13	1416.7
G14	2064.2
G15	1614.7
G16	1386.7
G17	1107.2
G18	1877.1
G19	636.7

Here, |*R_i_ - R_j_*| is the absolute difference in molar mass between adjacent residues (vertices Ri and Rj), and $\bar{d}$ is the average degree of the top-level graph, reflecting the typical number of connections per vertex [61]. This normalization by the average degree accounts for the structural density of the graph, providing a more comparable measure across different subdomains. The parameter *ΔMd* is critical to our study as it offers insight into the structural and chemical heterogeneity within the midlevel graphs. By measuring the molar mass variation between adjacent vertices relative to the graph’s connectivity, ΔMd helps us understand how mass distribution correlates with the network topology of the subdomains. This is particularly relevant for identifying regions of significant chemical or structural divergence, which may influence the functional properties of the system under study. For instance, a high ΔMd value could indicate a sharp transition in molecular composition across connected residues, potentially pointing to functionally important boundaries or interfaces within the system. Incorporating this analysis, therefore, supports our broader objective of mapping structural features to functional outcomes, as detailed in subsequent sections of our research.

### Virtual Mutations in SARS-CoV-2 Spike RBD (Chain E)

To evaluate the impact of single point mutations on the entire SARS-CoV-2 spike RBD corresponding to chain E of the structural protein, we selected 5 prevalent mutations associated with mild or severe COVID-19 from existing literature. Table 3 provides a comprehensive view of how each mutation influences the SARS-CoV-2 spike RBD corresponding to chain E of the structural protein.

**Table 3.** Mutation phenotypes of SARS-CoV-2 spike receptor-binding domain [102-106].

Mutation	Strain	Phenotype	Impact or clinical manifestation
Wildtype	Wuhan-Hu-1	Typical or normal form of a species as it occurs in nature; baseline for comparison with mutated or altered forms	No impact/baseline
K417N	Beta (B.1.351) Gamma (P.1) Omicron (BA.1) Omicron (BA.2)	Moderate	Immune evasion, associated with reduced neutralization by antibodies
N440K	Omicron (BA.1) Omicron (BA.2)	Mild	Potential immune escape, enhances binding to ACE2^a^ receptor but not a major antigenic change
L452R	Delta (B.1.617.2 and AY lineages) Kappa (B.1.617.1)	Severe	Immune evasion and transmission, linked to resistance to neutralizing antibodies and higher infectivity
E484A	Omicron	Moderate	Immune evasion, reduces neutralization by convalescent and vaccine-induced sera
N501Y	Alpha Beta Omicron Mu	Very severe	Increased binding affinity and immune escape, enhances ACE2 binding and is associated with immune evasion

a ACE2: angiotensin-converting enzyme 2.

Subsequently, the changes in molar mass between adjacent vertices per average degree (as shown in Table 2), derived from the weighted network interaction data, were assigned as vertex weights for the subdomain graphs within the top-level graph, G. This step enabled the generation of molecular descriptors (graph invariants) based on the weighted network interaction data in Cystoscape [92], representing the edge-interaction weights of the top-level graph. The resulting molecular database of graph invariants represented the wildtype graph (no mutation). For each of the 5 mutations, a virtual mutation process was performed. This involved identifying the specific amino acid in the relevant subdomain graph, mutating it via substitution or deletion, and creating a “mutant-specific vertex-weighted graph” for the affected subdomain by submitting the mutated FASTA sequence to I-TASSER [90,91] for ab initio modeling (see Multimedia Appendix 1 for mutant-specific vertex-weighted graphs). New graph-theoretic molecular descriptors were then computed for each mutated subdomain graph and applied to the top-level graph. Subsequently, the same molecular descriptors previously computed for the top-level graph of the wildtype were recalculated to capture the mutation’s impact. This approach allowed us to observe both local (subdomain) and global (entire SARS-CoV-2 spike RBD, corresponding to chain E of the structural protein) effects of each point mutation on the SARS-CoV-2 spike protein. Table 4 presents a comprehensive set of molecular descriptors for the wildtype SARS-CoV-2 spike RBD and its various mutations. These graph invariants offer quantitative insights into how mild and severe mutations affect the RBD’s structural and functional characteristics. Through analysis of these metrics across various variants, we elucidated the distinct effects of each mutation on the spike protein’s characteristics and functional implications.

**Table 4.** Edge (interaction) weights of mutation phenotypes for SARS-CoV-2 spike receptor-binding domain (chain E).

Subdomain interaction	Wildtype	K417N	N440K	L452R	N501Y	E484A
G14 (meta) G15	0.08990	0.08990	0.08990	0.08990	0.08990	0.10150
G13 (meta) G14	0.12950	0.12950	0.12950	0.12090	0.12950	0.12950
G12 (meta) G13	0.01126	0.01126	0.00844	0.01986	0.01126	0.01126
G11 (meta) G12	0.02020	0.02020	0.02302	0.02020	0.02020	0.02020
G7 (meta) G18	0.09210	0.09210	0.09210	0.09210	0.09210	0.09210
G2 (meta) G18	0.19724	0.19724	0.19724	0.19724	0.19724	0.19724
G11 (meta) G18	0.12354	0.12354	0.12354	0.12354	0.12354	0.12354
G12 (meta) G18	0.10334	0.10334	0.10052	0.10334	0.10334	0.10334
G9 (meta) G18	0.13476	0.13476	0.13476	0.13476	0.13476	0.13476
G4 (meta) G14	0.12532	0.12532	0.12532	0.12532	0.12532	0.12532
G3 (meta) G13	0.07532	0.07532	0.07532	0.08392	0.07532	0.07532
G3 (meta) G18	0.16740	0.16740	0.16740	0.16740	0.16740	0.16740
G3 (meta) G4	0.07950	0.07950	0.07950	0.07950	0.07950	0.07950
G2 (meta) G3	0.02984	0.02984	0.02984	0.02984	0.02984	0.02984
G3 (meta) G14	0.20482	0.20482	0.20482	0.20482	0.20482	0.20482
G11 (meta) G17	0.03044	0.03044	0.03044	0.03044	0.02062	0.03044
G12 (meta) G17	0.05064	0.05064	0.05346	0.05064	0.04082	0.05064
G9 (meta) G17	0.01922	0.01922	0.01922	0.01922	0.00940	0.01922
G17 (meta) G18	0.15398	0.15398	0.15398	0.15398	0.14416	0.15398
G8 (meta) G9	0.05224	0.05224	0.05224	0.05224	0.05224	0.05224
G8 (meta) G18	0.08252	0.08252	0.08252	0.08252	0.08252	0.08252
G3 (meta) G8	0.08488	0.08488	0.08488	0.08488	0.08488	0.08488
G4 (meta) G8	0.00538	0.00538	0.00538	0.00538	0.00538	0.00538
G8 (meta) G17	0.07146	0.07146	0.07146	0.07146	0.06164	0.07146
G8 (meta) G7	0.00958	0.00958	0.00958	0.00958	0.00958	0.00958
G6 (meta) G7	0.06766	0.06766	0.06766	0.06766	0.06766	0.06766
G6 (meta) G11	0.03622	0.03622	0.03622	0.03622	0.03622	0.03622
G8 (meta) G19	0.16556	0.16556	0.16556	0.16556	0.16556	0.16556
G7 (meta) G19	0.15598	0.15598	0.15598	0.15598	0.15598	0.15598
G4 (meta) G19	0.16018	0.16018	0.16018	0.16018	0.16018	0.16018
G19 (meta) G18	0.24808	0.24808	0.24808	0.24808	0.24808	0.24808
G1 (meta) G2	0.05444	0.05444	0.05444	0.05444	0.05444	0.05444
G1 (meta) G4	0.16378	0.16378	0.16378	0.16378	0.16378	0.16378
G5 (meta) G19	0.14654	0.14654	0.14654	0.14654	0.14654	0.14654
G5 (meta) G4	0.01364	0.01364	0.01364	0.01364	0.01364	0.01364
G1 (meta) G5	0.15014	0.15014	0.15014	0.15014	0.15014	0.15014
G5 (meta) G6	0.05822	0.05822	0.05822	0.05822	0.05822	0.05822
G5 (meta) G7	0.00944	0.00944	0.00944	0.00944	0.00944	0.00944
G16 (meta) G15	0.04560	0.04560	0.04560	0.04560	0.04560	0.03400
G12 (meta) G16	0.00526	0.00526	0.00244	0.00526	0.00526	0.00526
G13 (meta) G16	0.00600	0.00600	0.00600	0.01460	0.00600	0.00600
G16 (meta) G17	0.05590	0.05590	0.05590	0.05590	0.04608	0.05590
G14 (meta) G16	0.13550	0.13550	0.13550	0.13550	0.13550	0.13550
G9 (meta) G16	0.03668	0.03668	0.03668	0.03668	0.03668	0.03668
G8 (meta) G10	0.10574	0.10292	0.10574	0.10574	0.10574	0.10574
G9 (meta) G10	0.15798	0.15516	0.15798	0.15798	0.15798	0.15798
G10 (meta) G14	0.01420	0.01702	0.01420	0.01420	0.01420	0.01420
G10 (meta) G11	0.14676	0.14394	0.14676	0.14676	0.14676	0.14676
G3 (meta) G10	0.19062	0.18780	0.19062	0.19062	0.19062	0.19062
G10 (meta) G13	0.11530	0.11248	0.11530	0.10670	0.11530	0.11530

### Unsupervised Machine Learning Analysis of Point Mutations Associated With SARS-CoV-2 Spike RBD (Chain E)

To analyze the impact of point mutations associated with SARS-CoV-2 spike RBD (chain E), we used our previous novel approach combining graph theory and machine learning [59,84,85]. Our method used graph-theoretic molecular-weighted invariants or descriptors, as detailed in Table 4, for the wildtype SARS-CoV-2 spike RBD (chain E) and mild and severe mutations associated with SCD. We applied an unsupervised machine learning technique, specifically hierarchical clustering, to visualize the variations between each SARS-CoV-2 spike RBD (chain E) point mutation and the wildtype SARS-CoV-2 spike RBD (chain E). Hierarchical clustering provided insights into the structure and relationships within the datasets. The analysis was conducted using Python statistical software (Python Software Foundation), using the single linkage function and Euclidean distance without setting a predefined number of clusters or distance threshold to generate a dendrogram for the SARS-CoV-2 spike RBD mutation phenotypes. We opted for the single-linkage function to minimize potential biases in clustering the SARS-CoV-2 spike RBD (chain E)–related point mutations [100,107]. This approach allowed us to create a visual representation of how virtual SARS-CoV-2 spike RBD (chain E)–related point mutations affected the entire SARS-CoV-2 spike RBD (chain E) structure in comparison to the wildtype. Through this approach, we were able to distinguish how the different virtual mutations differed from the wildtype, providing an insight into how the point mutations impacted the entire SARS-CoV-2 spike RBD.

### Ab Initio Modeling of Mutated Protein Sequences Using I-TASSER

To further elucidate the structural and functional implications of spike protein mutations, we implemented an ab initio modeling approach using the I-TASSER platform [90]. This state-of-the-art predictive modeling tool was used to generate high-resolution, 3D structural models of the spike protein variants [90,91,101]. The I-TASSER algorithm uses a hierarchical approach, combining threading, fragment assembly, and atomic-level structure refinement to predict protein structure and function. We input the mutated spike protein sequences into the I-TASSER server [92], which then produced detailed structural models. These models were analyzed to identify potential alterations in protein folding, binding site configurations, and overall conformational changes resulting from the point mutations. The visual representations derived from this process provided crucial insights into the molecular-level effects of the mutations, complementing our graph-theoretic modeling approach. By integrating these computational methodologies, we were able to establish a comprehensive framework for understanding the relationship between sequence-level mutations and their macromolecular consequences, offering a multifaceted view of the spike protein’s structural and functional adaptations.

### MD Simulations of Wildtype and Mutated Proteins in Water

To complement our findings from graph-theoretic modeling, we investigated whether MD simulations could effectively replicate the effects of point mutations leading to diverse phenotypes among the COVID-19 mutations analyzed in this study. MD simulations have emerged as a critical tool for elucidating the structural and functional implications of protein mutations, specifically in the context of the SARS-CoV-2 spike protein. In this work, we focused exclusively on the spike protein, both in its wildtype form and mutated variants, without the inclusion of the hACE2 receptor or other interacting proteins. This approach allowed us to isolate the intrinsic dynamic behavior and stability of the spike protein under varying mutational conditions. The dynamic insights gained from these simulations have proven instrumental in designing stabilized S2 immunogens for SARS-CoV-2, which demonstrate enhanced protein expression, superior thermostability, and preserved immunogenicity against sarbecoviruses.

The MD simulations were conducted using the WebGRO for Macromolecular Simulations server [108], which operates on the GROMACS simulation package [102]. This platform was selected for its intuitive interface and capability to perform fully solvated MD simulations. The wildtype spike protein structures were obtained from the PDB, while mutated variants were generated using I-TASSER [90]. Both wildtype and mutated structures were prepared in orthorhombic simulation boxes, solvated with simple point charge (SPC) water, counterions, and 0.15 M NaCl to mimic physiological conditions. This setup was designed to closely replicate the biological environment, ensuring accurate observations of protein behavior in a dynamic state.

Before the production runs, energy minimization was carried out using the steepest descent integrator to eliminate steric clashes or unfavorable contacts within the system. This step is essential to position the system at a local energy minimum, a prerequisite for stable MD simulations. Subsequently, the system was equilibrated at 300 K and 1.1023 bar, reflecting the temperature and pressure conditions of the human body [103,109]. Position restraints on protein atoms were applied during the initial equilibration phase to stabilize the system before full MD production runs were initiated.

To ensure the robustness and reliability of our findings, triplicate simulations were performed at 3 distinct timescales: 50 nanoseconds, 100 nanoseconds, and 200 nanoseconds. Each simulation incorporated varied random sampling seed inputs to account for stochastic variations in the system’s dynamics. This methodology enabled the capture of both short-term fluctuations and long-term stability trends, providing a comprehensive view of the dynamic behavior of the spike protein over extended periods.

Protein interactions were modeled using the Optimized Potentials for Liquid Simulations–All Atom (OPLS-AA) force field, recognized for its precision in simulating protein-water interactions. The SPC/extended (SPC/E) water model was chosen for its ability to accurately represent the properties of water, particularly its dielectric constant and density, which are critical for realistic solvation dynamics. This combination of force field and water model ensured a high-fidelity representation of the protein’s environment during simulations.

Postsimulation analyses were conducted to evaluate the stability and conformational changes of the spike protein trajectories. Root mean square deviation (RMSD) was calculated to assess trajectory stability and deviations in atomic positions over time. RMSD plots provided visual insights into how much the protein structure deviated from its initial configuration, shedding light on conformational stability. Statistical metrics, including minimum and maximum RMSD values, SDs, and SEs, were computed to compare the stability between wildtype and mutated variants. In addition, the Kolmogorov-Smirnov test was applied to determine whether mutations significantly altered the distribution of protein conformations relative to the wildtype. Visualization and detailed analysis of trajectories were performed using VMD [104,110] and BIOVIA Discovery Studio (Dassault Systèmes BIOVIA, Discovery Studio Modeling Environment, Release 2017; Dassault Systèmes), enabling an in-depth examination of conformational changes and protein-water interactions. The simulations revealed dynamic insights into how mutations influence the stability of the spike protein, particularly with respect to conformational flexibility and hydration patterns around active sites, as observed through bundled SPC water models. These differences in hydration could potentially impact enzymatic activity or antigenicity.

The methodologies used in these MD simulations, from system setup to detailed postsimulation analysis, highlight the value of computational approaches in complementing experimental data. By focusing solely on the spike protein and excluding interactions with hACE2, we obtained a clearer understanding of the intrinsic effects of mutations on protein structure and dynamics. These insights are vital for structure-based vaccine design, as understanding the dynamic behavior of the spike protein can guide the development of vaccines targeting stable, immunogenic conformations. Ultimately, the MD simulations adopted in this study provide a powerful lens through which the effects of mutations on protein function can be observed in a dynamic, physiological context, contributing to a deeper comprehension of how mutations in the SARS-CoV-2 spike protein may influence its behavior and interaction with the host immune system.

## Results

### Graph-Theoretic Modeling Reveals Impact of Point Mutations on SARS-CoV-2 Spike RBD

We developed a 3-level weighted hierarchical graph-theoretic model of the SARS-CoV-2 spike RBD corresponding to chain E (see Figure 8). The model consisted of a foundation level with 20 vertex-weighted amino acids, a middle level with 19 vertex-weighted subdomain graphs, and a top level where subdomain graphs are consolidated into single weighted vertices. Key features of the model included vertex connections established using a 6-angstrom proximity threshold, subdomain graph vertex weights based on molar mass changes between adjacent vertices per average degree, and a virtual mutation process applied for 5 specific mutations (K417N, N440K, L452R, N501Y, and E484A). Mutant-specific vertex-weighted graphs were created using I-TASSER [90] for ab initio modeling, and new graph-theoretic molecular descriptors were computed for mutated subdomains and applied to the top-level graph. This approach enabled observation of both local (subdomain) and global (entire RBD) effects of each point mutation on the SARS-CoV-2 spike protein, facilitating understanding of how point mutations lead to different COVID-19 phenotypes. Hierarchical clustering analysis of SARS-CoV-2 spike protein variants was performed in Python using interaction data imported from a CSV file (see Multimedia Appendix 1) via the pandas library [111]. The data matrix was transposed to organize protein variants as rows, and clustering was conducted with the scipy.cluster.hierarchy.linkage function [112], applying the Euclidean distance metric and single linkage method to determine pairwise similarities. Variant labels included their respective mutated phenotypes for clarity. The dendrogram was visualized using matplotlib with bold axis labels and tick marks, and x-axis labels were rotated for readability. The figure was rendered at 300 dpi.

**Figure 9.**
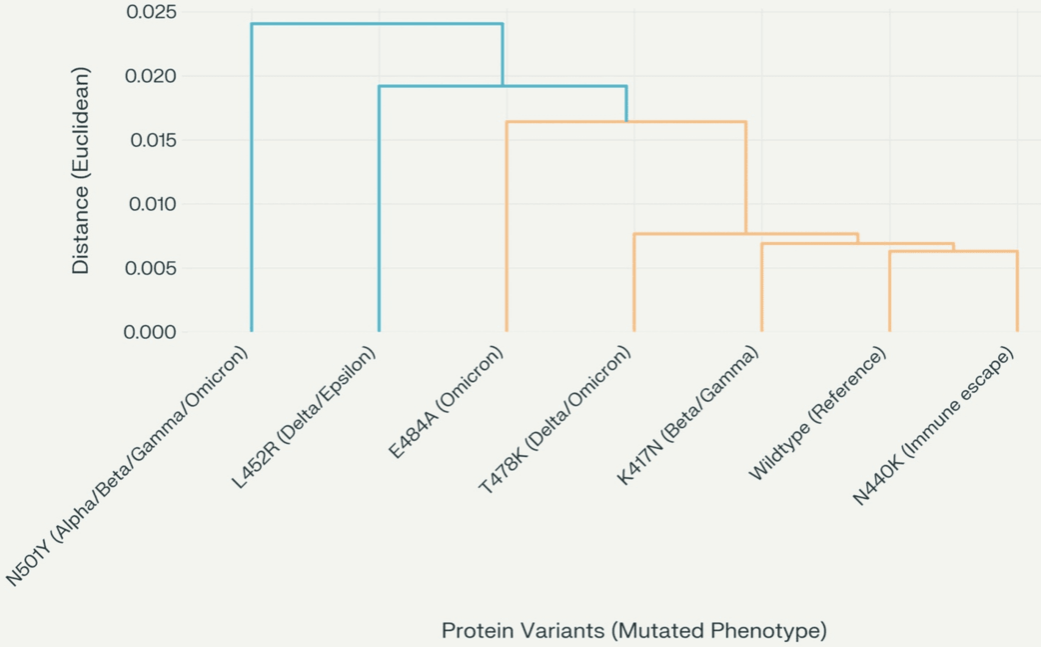
Clustering analysis of COVID-19 mutation-associated point mutations in SARS-CoV-2 receptor-binding domain. The hierarchical clustering dendrogram of SARS-CoV-2 spike protein variants based on interaction data (Table 4). Clustering was performed using the Euclidean distance metric and single linkage method. Each tip on the x-axis is labeled with the protein variant and its associated mutated phenotype: N501Y (Alpha/Beta/Gamma/Omicron), L452R (Delta/Epsilon), E484A (Omicron), T478K (Delta/Omicron), K417N (Beta/Gamma), wildtype (Reference), and N440K (Immune escape). The y-axis represents the Euclidean distance, indicating the degree of dissimilarity between variants. Both axis labels and tick marks are displayed in bold for clarity. The dendrogram reveals that N501Y and L452R form a distinct cluster, reflecting greater divergence from the other variants, while wildtype, K417N, and N440K group together, indicating higher similarity in their interaction profiles. This clustering highlights the structural and functional relationships among spike protein variants and their phenotypic classifications, providing insights into how specific mutations may influence SARS-CoV-2 spike protein network architecture. The clustering analysis was performed using Python with the scipy.cluster.hierarchy.linkage function [111], applying the Euclidean distance metric and single linkage method to determine pairwise similarities.

Figure 9 displays the resulting dendrogram, constructed from graph-theoretic descriptors (see Table 4 and the Methods section), providing a visual summary of how each virtual point mutation affects the SARS-CoV-2 spike RBD (chain E) relative to the wildtype. This clustering diagram highlights the structural and functional relationships among the mutated variants and the original wildtype RBD.

Dendrogram analysis (see Figure 8) revealed varying degrees of divergence between the wildtype and the analyzed mutations based on Euclidean distance. The severe N501Y mutation, characterized by the substitution of asparagine with tyrosine at position 501, showed the greatest divergence from the wildtype, with an approximate Euclidean distance of 0.023. The L425R mutation, involving the replacement of leucine with a positively charged arginine at position 425, also exhibited a significant difference, with a Euclidean distance of approximately 0.019. Similarly, the E484A mutation, where glutamic acid is substituted with alanine at position 484, demonstrated a divergence of approximately 0.017 Euclidean distance from the wildtype. In contrast, the K147N mutation, which replaces lysine with asparagine at position 147 within the N-terminal domain, was closer to the wildtype but still distinct, with an approximate Euclidean distance of 0.007. These results underscore the structural and functional variability introduced by these mutations relative to the wildtype.

### Ab Initio Models Reveal Structural Alterations in Mutated SARS-CoV-2 Spike RBD

**Figure 10.**
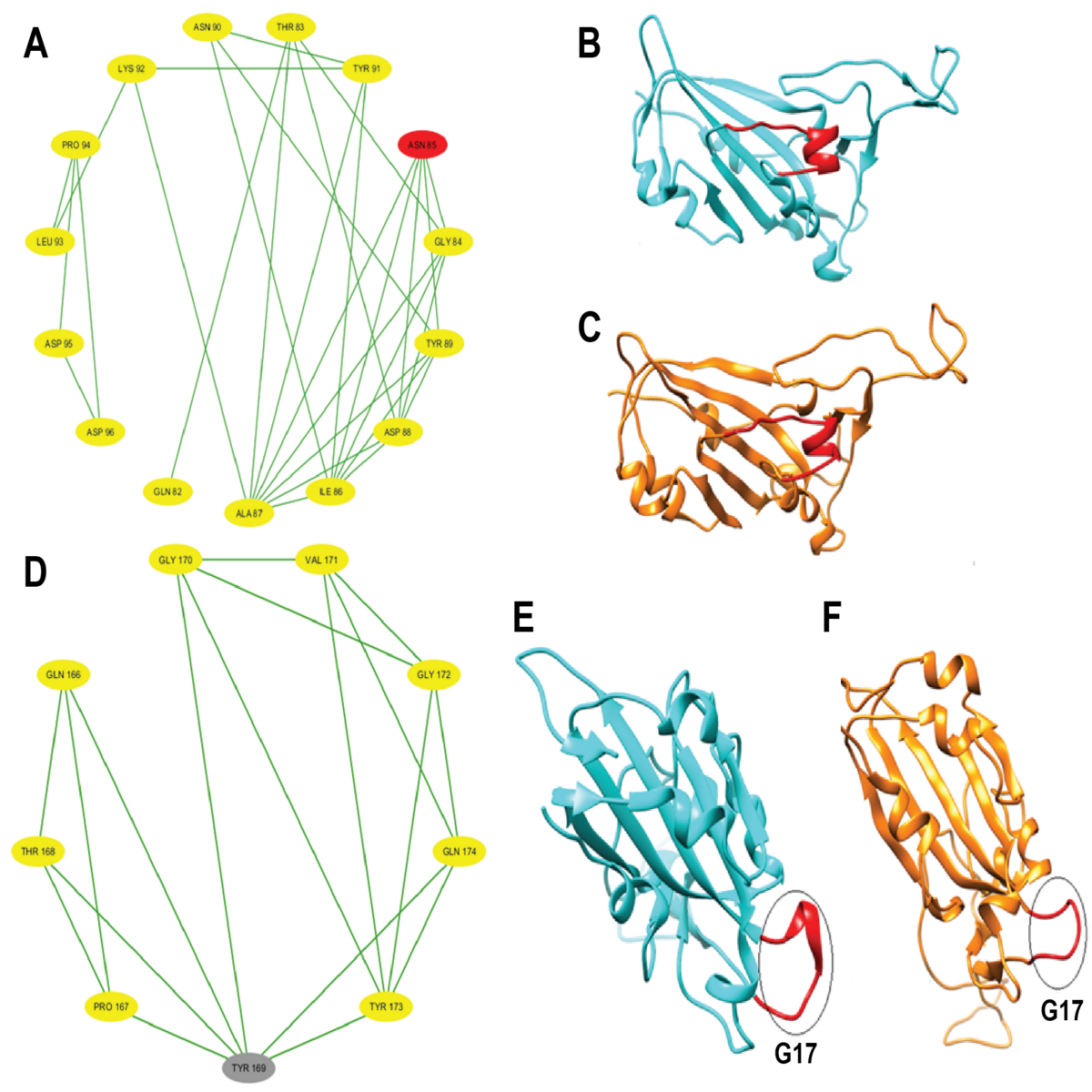
Local and global effects of mutations E484A and L452R on the SARS-CoV-2 spike receptor-binding domain (RBD; chain E) compared to wildtype. (A) Mutated subdomain graph G15 illustrating the mild E484A mutation. (B) Ab initio model of wildtype SARS-CoV-2 spike RBD (chain E). (C) Ab initio model of SARS-CoV-2 spike RBD (chain E) following the mild E484A mutation. (D) Mutated subdomain graph G13 depicting the severe L452R mutation. (E) Ab initio model of wildtype SARS-CoV-2 spike RBD (chain E). (F) Ab initio model of SARS-CoV-2 spike RBD (chain E) after the severe L452R mutation, illustrating significant alterations in protein folding and structural integrity.

**Figure 11.**
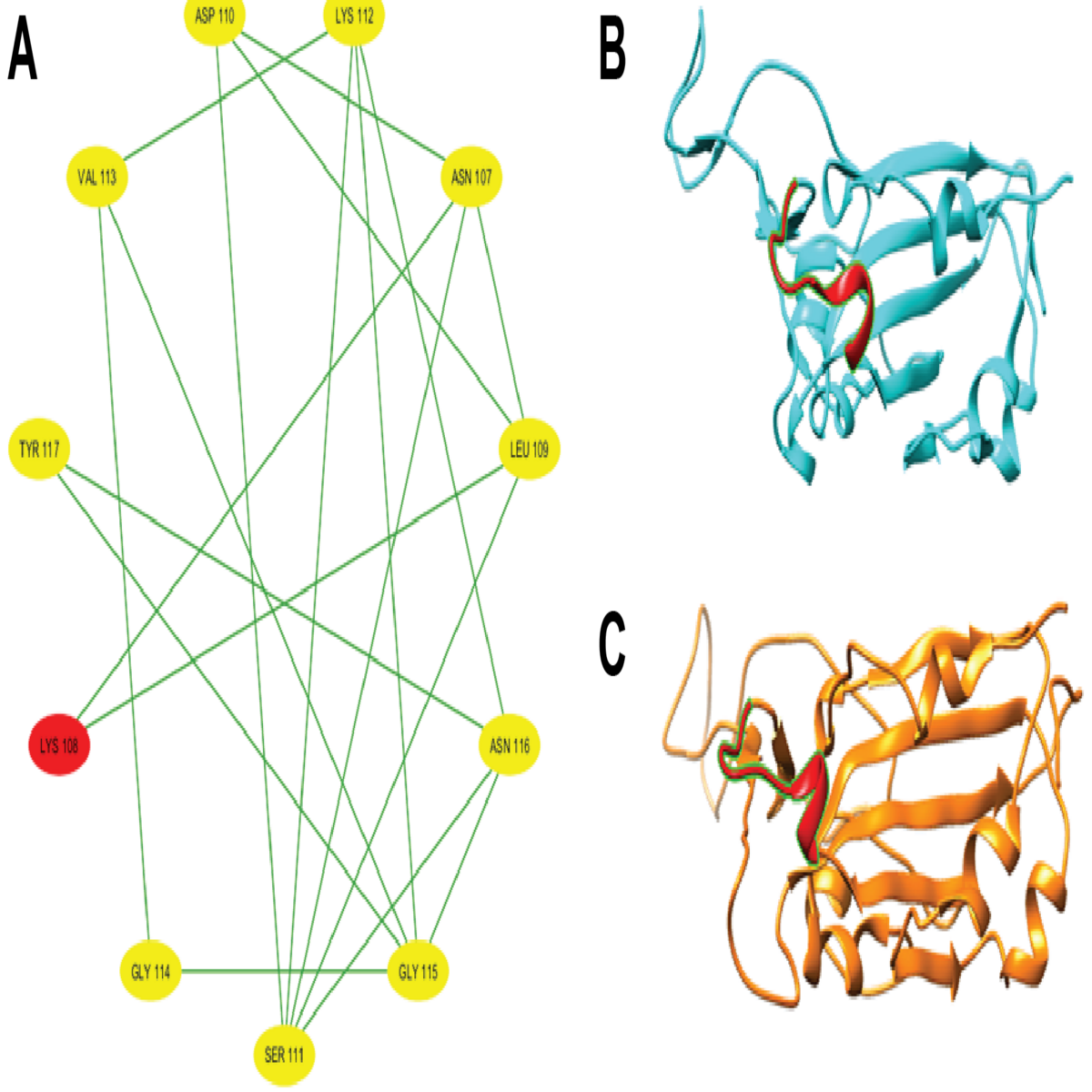
Local and global effects of mutations K417N and N501Y on the SARS-CoV-2 spike receptor-binding domain (RBD; chain E) compared to wildtype. (A) Mutated subdomain graph G10 illustrating the mild K417N mutation. (B) Ab initio model of wildtype SARS-CoV-2 spike RBD (chain E). (C) Ab initio model of SARS-CoV-2 spike RBD (chain E) following the mild K417N mutation. (D) Mutated subdomain graph G17 depicting the severe N501Y mutation. (E) Ab initio model of wildtype SARS-CoV-2 spike RBD (chain E). (F) Ab initio model of SARS-CoV-2 spike RBD (chain E) after the N501Y mutation, illustrating significant alterations in protein folding and structural integrity.

**Figure 12.**
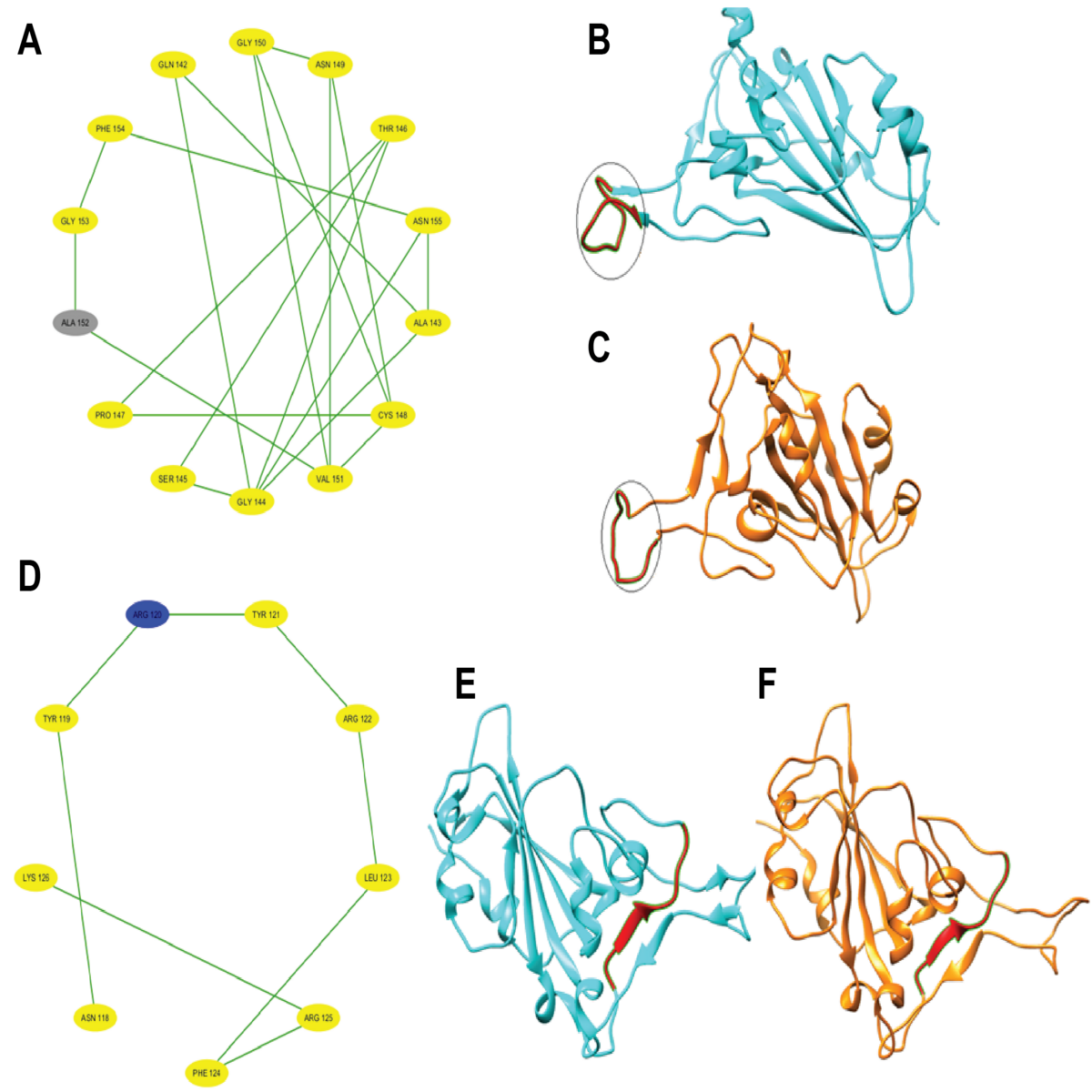
Local and global effects of mutation N440K on the SARS-CoV-2 spike receptor-binding domain (RBD; chain E) compared to wildtype. (A) Mutated subdomain graph G12 illustrating the mild N440K mutation. (B) Ab initio model of wildtype SARS-CoV-2 spike RBD (chain E). (C) Ab initio model of SARS-CoV-2 spike RBD (chain E) following the mild N440K mutation.

To gain deeper insights into the impact of COVID-19–specific mutations on the SARS-CoV-2 spike RBD (chain E) protein conformation, ab initio models were generated using I-TASSER [92] and visualized in Cystoscape [96]. These models provide a comparative view of selected point mutations (N440K, E484A, N501Y, K417N, and L452R) against the wildtype structure. Figures 10-12 illustrate the structural changes induced by each mutation, revealing that even a seemingly mild mutation like E484A can significantly affect secondary structures and global protein folding. The models demonstrate alterations in protein folding patterns, changes in structural integrity, and modifications to local and global conformations. These visualizations highlight the profound impact of mutations on the SARS-CoV-2 spike RBD, emphasizing how small changes can lead to substantial structural rearrangements. Such alterations may influence the virus’s infectivity, immune evasion, and interaction with the ACE2 receptor. Notably, the N501Y mutation shows significant local and regional changes in the ab initio model, affecting protein folding and structural integrity. These modifications are more pronounced when compared to the wildtype structure, indicating that the N501Y mutation substantially impacts the RBD’s conformation as shown by the ab initio modeling results in Figure 11F. The E484A mutation, despite appearing mild, demonstrates detrimental effects on secondary structures and global protein folding (see Figure 10A). Other mutations (N440K, K417N, and L452R) exhibit varying degrees of impact on the RBD structure (see Figures10D, 11A,  12), potentially influencing the spike protein’s function and stability. These structural insights provide a foundation for understanding the molecular mechanisms behind the enhanced transmissibility and potential immune evasion of SARS-CoV-2 variants, contributing to our knowledge of the virus’ evolution and informing future therapeutic strategies.

### MD Simulation Analysis Unveils Differential Structural Stability Between Wildtype and Mutated SARS-CoV-2 Spike Proteins

To complement our graph-theoretic and ab initio modeling findings, we conducted MD simulations to investigate the effects of point mutations on COVID-19 variants. These simulations provided insights into the binding interactions and stability of wildtype and mutated SARS-CoV-2 spike proteins in dynamic states. Our approach aligns with recent studies that have used MD simulations to examine the conformational behavior of SARS-CoV-2 spike protein variants.

We utilized the WebGRO server [108], based on GROMACS [109], to prepare modeled structures in orthorhombic simulation boxes. The proteins were solvated with SPC water, counterions, and 0.15 M NaCl to mimic physiological conditions. To maintain consistency, only the spike protein and the mutated spike proteins were used in the protein-in-water simulation studies, mirroring the approach used in the graph-theoretic and ab initio modeling. Energy minimization was performed using the steepest descent integrator, followed by equilibration at 300 K and 1.1023 bar. For robustness, we conducted triplicate 50-, 100-, and 200-nanosecond simulations with varied random sampling seed inputs. Both wildtype protein structures from PDB and mutated variants generated via I-TASSER [92,98] were simulated using the OPLS-AA force field and SPC/E water models.

**Figure 13.**
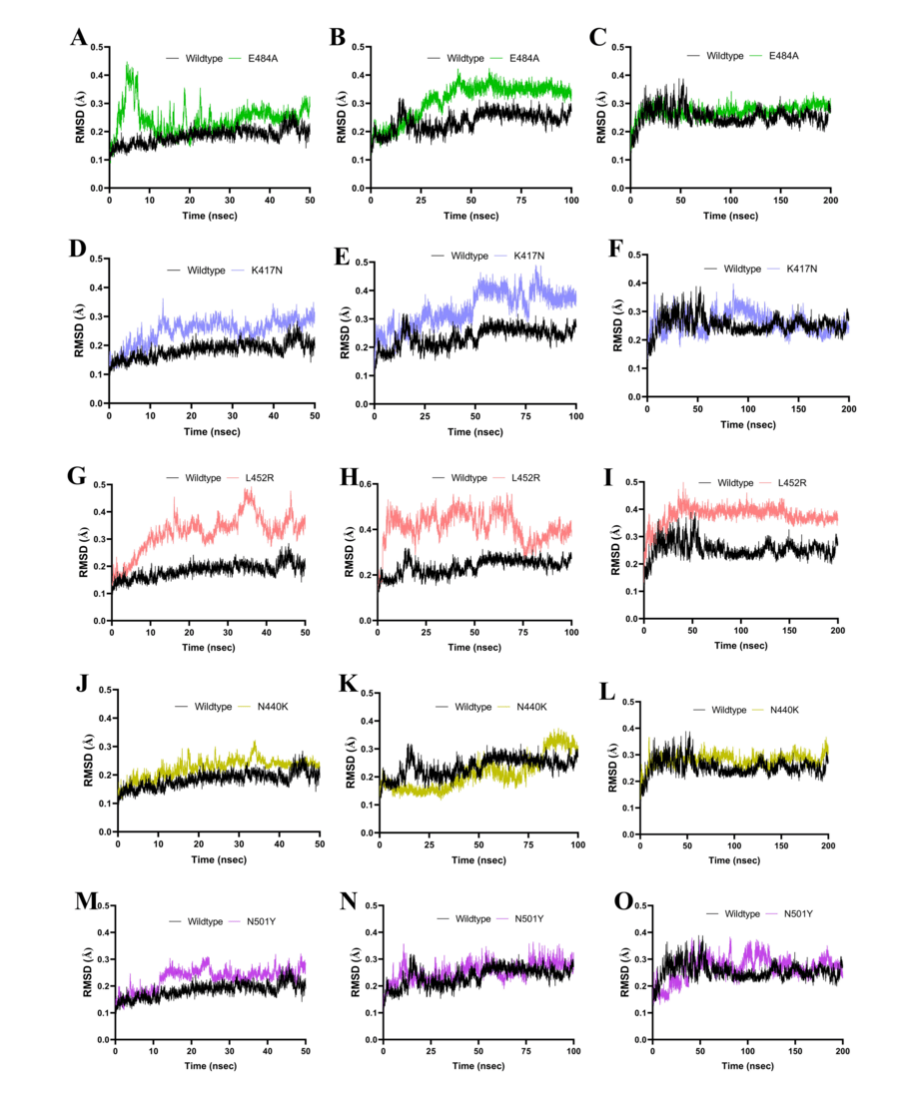
Molecular dynamics simulations reveal timescale-dependent differential structural stability of wildtype and mutated SARS-CoV-2 spike proteins. Root mean square deviation (RMSD) analysis was performed on wildtype (black) and mutated spike proteins—E484A (green), K417N (lavender), L452R (pink), N440K (olive), and N501Y (indigo)—across short (50 ns), intermediate (100 ns), and long (200 ns) timescales. Each panel displays the RMSD trajectory for wildtype and a specific mutant at a given timescale: E484A (green) in panels (A) 50 ns (RMSD range 4.00×10^-7^ to 0.4477065; mean 0.2435, standard error of the mean [SEM] 0.000727), (B) 100 ns (RMSD range 4.00×10^-7^ to 0.4236228; mean 0.3108, SEM 0.000896), and (C) 200 ns (RMSD range 4.00×10^-7^ to 0.4477065; mean 0.2435, SEM 0.000727); K417N (lavender) in panels (D) 50 ns (RMSD range 5.00×10^-7^ to 0.3620131; mean 0.2492, SEM 0.000624), (E) 100 ns (RMSD range 4.00×10^-7^ to 0.4897364; mean 0.3362, SEM 0.000897), and (F) 200 ns (RMSD range 5.00×10^-7^ to 0.3620131; mean 0.2492, SEM 0.000624); L452R (pink) in panels (G) 50 ns (mean 0.3224, SEM 0.000984), (H) 100 ns (RMSD range 5.00×10^-7^ to 0.561134; mean 0.4151, SEM 0.000957), and (I) 200 ns (mean 0.3224, SEM 0.000984); N440K (olive) in panels (J) 50 ns (RMSD range 5.00×10^-7^ to 0.3220472; mean 0.2230, SEM 0.00048), (K) 100 ns (RMSD range 1.10×10^–6^ to 0.3744204; mean 0.2123, SEM 0.000862), and (L) 200 ns (RMSD range 4.00×10^-7^ to 0.367109; mean 0.2796, SEM 0.000353); N501Y (indigo) in panels (M) 50 ns (RMSD range 4.00×10^-7^ to 0.320323; mean 0.2279, SEM 0.000559), (N) 100 ns (RMSD range 3.00×10^-7^ to 0.3636647; mean 0.2529, SEM 0.000484), and (O) 200 ns (RMSD range 4.00×10^-7^ to 0.320323; mean 0.2279, SEM 0.000559); and wildtype (black) with RMSD values of 50 ns (RMSD range 3.00×10^-7^ to 0.284101; mean 0.1838, SEM 0.000381), 100 ns (RMSD range 3.00×10^-7^ to 0.3198064; mean 0.2351, SEM 0.000495), and 200 ns (RMSD range 3.00×10^-7^ to 0.284101; mean 0.1838, SEM 0.000381). The 2-sample Kolmogorov-Smirnov tests confirmed significant conformational deviations for all mutations compared to wildtype (*P*<2.2×10^–16^; α=.05), except for N440K (*P*=1.43×10^–232^) and N501Y (*P*=1.18×10^–64^) at the 100-ns timescale. L452R consistently exhibited the highest RMSD means among all mutants, especially at the intermediate timescale, while N440K showed the lowest RMSD mean among mutants at 100 ns. For N440K at 200 ns, the RMSD ranged from 4.00×10^-7^ to 0.367109, with a mean of 0.2796 (SEM 0.000353) and a Kolmogorov-Smirnov test *P* value of 2.20×10^–16^, indicating significant structural deviation from wildtype. These results highlight mutation and timescale-dependent destabilization of the spike protein structure.

Postsimulation analyses focused on RMSD plots to assess trajectory stability and atomic position deviations. Visualization and analysis of trajectories were performed using VMD and BIOVIA Discovery Studio. This comprehensive approach enabled detailed comparisons of protein-water interactions and stability across variants, providing valuable insights into the effects of mutations on protein behavior in dynamic environments. As illustrated in Figure 13, RMSD analysis revealed differential structural stability between wildtype and mutated SARS-CoV-2 spike proteins.

**Figure 14. F14:**
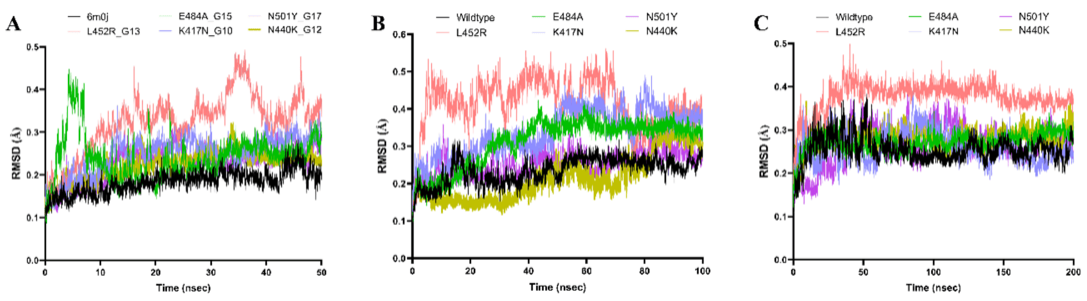
Overlaid root mean square deviation (RMSD) plots illustrate the conformational stability of wildtype (black) and mutated SARS-CoV-2 spike proteins—E484A (green), K417N (lavender), L452R (pink), N440K (olive), and N501Y (indigo)—across molecular dynamics simulation timescales of 50 ns, 100 ns, and 200 ns. At 50 ns, mean (standard error of the mean [SEM]) values for WT, E484A, K417N, L452R, N440K, and N501Y were 0.1838 (SEM 0.000381), 0.2435 (SEM 0.000727), 0.2492 (SEM 0.000624), 0.3224 (SEM 0.000984), 0.2230 (SEM 0.000480), and 0.2279 (SEM 0.000559) Å, respectively. At 100 ns, values were mean 0.2351 (SEM 0.000495) for WT, mean 0.3108 (SEM 0.000896) for E484A, mean 0.3362 (SEM 0.000897) for K417N, mean 0.4151 (SEM 0.000957) for L452R, mean 0.2123 (SEM 0.000862) for N440K, and mean 0.2529 (SEM 0.000484) for N501Y. At 200 ns, WT, E484A, K417N, L452R, N440K, and N501Y had mean (SEM) values of 0.1838 (SEM 0.000381), 0.2435 (SEM 0.000727), 0.2492 (SEM 0.000624), 0.3224 (SEM 0.000984), 0.2796 (SEM 0.000353, SD 0.02494), and 0.2279 (SEM 0.000559) Å, respectively. L452R demonstrates the highest RMSD means across all timepoints, indicating pronounced instability, while N440K shows the lowest mean among mutants at 100 ns but increased deviation at 200 ns. These overlaid RMSD trajectories highlight mutation- and timescale-dependent conformational changes in the spike protein structure.

[Fig F14]In Figure 13, RMSD analysis shows timescale-dependent structural stability for wildtype and mutated SARS-CoV-2 spike proteins. The wildtype (black) consistently exhibited stability at 50 nanoseconds (range 3×10^–7^ to 0.2841; mean 0.1838, standard error of the mean [SEM] 0.000381), 100 nanoseconds (range 3×10^–7^ to 0.3198; mean 0.2351, SEM 0.000495), and 200 nanoseconds (range 3×10^–7^ to 0.2841; mean 0.1838, SEM 0.000381). Mutants showed increased instability: E484A (green) had 50 nanoseconds (range 4×10^–7^ to 0.4477; mean 0.2435, SEM 0.000727), 100 nanoseconds (range 4×10^–7^ to 0.4236; mean 0.3108, SEM 0.000896), and 200 nanoseconds (range 4×10^–7^ to 0.4477; mean 0.2435, SEM 0.000727); K417N (lavender) had 50 nanoseconds (range 5×10^–7^ to 0.3620; mean 0.2492, SEM 0.000624), 100 nanoseconds (range 4×10^–7^ to 0.4897; mean 0.3362, SEM 0.000897), and 200 nanoseconds (range 5×10^–7^ to 0.3620; mean 0.2492, SEM 0.000624); L452R (pink) showed a mean of 0.3224 (SEM 0.000984) at 50 nanoseconds and 200 nanoseconds, and at 100 nanoseconds (range 5×10^–7^ to 0.5611; mean 0.4151, SEM 0.000957); N440K (olive) had 50 nanoseconds (range 5×10^–7^ to 0.3220; mean 0.2230, SEM 0.000480), 100 nanoseconds (range 1.10×10^–6^ to 0.3744; mean 0.2123, SEM 0.000862), and 200 nanoseconds (range 4×10^–7^ to 0.3671; mean 0.2796, SEM 0.000353, SD 0.02494), with the Kolmogorov-Smirnov test confirming significant deviation from wildtype at 200 nanoseconds (*P*=2.20×10^–16^); N501Y (indigo) had 50 nanoseconds (range 4×10^–7^ to 0.3203; mean 0.2279, SEM 0.000559), 100 nanoseconds (range 3×10^–7^ to 0.3637; mean 0.2529, SEM 0.000484), and 200 nanoseconds (range 4×10^–7^ to 0.3203; mean 0.2279, SEM 0.000559). Statistical tests confirmed significant conformational deviation for most mutations compared to wildtype (*P*<2.2×10^–16^; α=.05), except for N440K (*P*=1.43×10^–23^) and N501Y (*P*=1.18×10^–64^) at 100 ns. L452R exhibited the highest mean RMSD especially at 100 nanoseconds, while N440K showed the lowest among mutants at 100 nanoseconds, highlighting mutation- and timescale-dependent destabilization. Figure 14 presents corresponding RMSD trajectories.

In Figure 13, RMSD analysis shows timescale-dependent structural stability for wildtype and mutated SARS-CoV-2 spike proteins. The wildtype (black) consistently exhibited stability at 50 nanoseconds (range 3×10^–7^ to 0.2841; mean 0.1838, SEM 0.000381), 100 nanoseconds (range 3×10^–7^ to 0.3198; mean 0.2351, SEM 0.000495), and 200 nanoseconds (range 3×10^–7^ to 0.2841; mean 0.1838, SEM 0.000381). Mutants showed increased instability: E484A (green) had 50 nanoseconds (range 4×10^–7^ to 0.4477; mean 0.2435, SEM 0.000727), 100 nanoseconds (range 4×10^–7^ to 0.4236; mean 0.3108, SEM 0.000896), and 200 nanoseconds (range 4×10^–7^ to 0.4477; mean 0.2435, SEM 0.000727); K417N (lavender) had 50 nanoseconds (range 5×10^–7^ to 0.3620; mean 0.2492, SEM 0.000624), 100 nanoseconds (range 4×10^–7^ to 0.4897; mean 0.3362, SEM 0.000897), and 200 nanoseconds (range 5×10^–7^ to 0.3620; mean 0.2492, SEM 0.000624); L452R (pink) showed a mean of 0.3224 (SEM 0.000984) at 50 nanoseconds and 200 nanoseconds, and at 100 ns (range 5×10^–7^ to 0.5611; mean 0.4151, SEM 0.000957); N440K (olive) had 50 nanoseconds (range 5×10^–7^ to 0.3220; mean 0.2230, SEM 0.000480), 100 nanoseconds (range 1.10×10^–6^ to 0.3744; mean 0.2123, SEM 0.000862), and 200 nanoseconds (range 4×10^–7^ to 0.3671; mean 0.2796, SEM 0.000353, SD 0.02494), with the Kolmogorov-Smirnov test confirming significant deviation from wildtype at 200 nanoseconds (*P*=2.20×10^–16^); N501Y (indigo) had 50 nanoseconds (range 4×10^–7^ to 0.3203; mean 0.2279, SEM 0.000559), 100 nanoseconds (range 3×10^–7^ to 0.3637; mean 0.2529, SEM 0.000484), and 200 nanoseconds (range 4×10^–7^ to 0.3203; mean 0.2279, SEM 0.000559). Statistical tests confirmed significant conformational deviation for most mutations compared to wildtype (*P*<2.2×10^–16^; α=.05), except for N440K (*P*=1.43×10^–2^) and N501Y (*P*=1.18×10^–64^) at 100 nanoseconds. L452R exhibited the highest mean RMSD especially at 100 nanoseconds, while N440K showed the lowest among mutants at 100 nanoseconds, highlighting mutation- and timescale-dependent destabilization; Figure 14 presents corresponding RMSD trajectories.

## Discussion

### Principal Findings

The dendrogram analysis (see Figure 9) highlights significant structural and functional differences between several mutations and the wildtype SARS-CoV-2 spike protein, particularly the N501Y, L452R, E484A, and K147N mutations. These differences, quantified by Euclidean distances from the wildtype, reflect the profound alterations induced by these mutations. The N501Y mutation (asparagine to tyrosine at position 501) and L452R mutation (leucine to arginine at position 452) exhibit substantial deviations from the wildtype, with Euclidean distances of approximately 0.023 and 0.019, respectively. These mutations significantly impact the spike RBD, enhancing infectivity and immune evasion. As illustrated in Figure 8, these mutations are distinctly clustered within the dendrogram, highlighting their significant divergence from the wildtype. The notable Euclidean distances (N501Y: 0.023; L452R: 0.019) indicate substantial structural alterations, which correspond to their clinical relevance in enhancing viral transmission and facilitating immune escape. Specifically, the N501Y mutation increases binding affinity to the hACE2 receptor, enhancing viral transmissibility and infectivity. It has been linked to VOCs, such as Alpha, Beta, and Gamma, and enables infection across a broader range of hosts, including mice. This mutation represents a critical adaptive change in SARS-CoV-2 evolution, underscoring its role in the pandemic [28,100]. Similarly, the L452R mutation introduces a positively charged arginine in place of leucine within a hydrophobic region of the RBD. This disrupts local hydrophobic interactions and destabilizes the protein structure, contributing to immune evasion and enhanced infectivity. The dendrogram clustering (see Figure 8) underscores its significant impact, consistent with clinical observations of resistance to neutralizing antibodies and increased viral transmission [101,102].

The E484A mutation (glutamic acid to alanine at position 484) and the K147N mutation (lysine to asparagine at position 147) also exhibit deviations from the wildtype, with Euclidean distances of approximately 0.017 and 0.0075, respectively. Although these mutations show less pronounced structural alterations compared to N501Y and L452R, as indicated by their Euclidean distances in Figure 9, they still represent significant changes in protein structure and function, as illustrated in Figure 9. Research has demonstrated that the E484A mutation impairs antibody recognition, enhancing immune evasion [103]. Similarly, the K147N mutation, located in the N-terminal domain, reduces neutralization by antibodies [104,105]. Although their effects are milder compared to N501Y and L452R, these findings highlight that even seemingly minor mutations can induce important structural changes with functional consequences.

Our study used I-TASSER [90] models and Cytoscape visualizations to investigate structural changes in mutated RBDs of the SARS-CoV-2 spike protein. The analysis revealed significant alterations in the mutated RBDs compared to the wildtype, both at the local mutation sites and in the overall RBD conformation. These findings suggest potential long-range effects on protein dynamics. Our visualizations highlighted localized changes in protein folding near mutation sites and potential impacts on the overall stability and flexibility of the RBD. These structural changes provide a molecular basis for understanding the observed impacts on binding affinity and dynamics of the spike protein. A prime example of these effects is the N501Y mutation in the SARS-CoV-2 spike RBD, known to enhance ACE2 receptor binding. Our study showed that this mutation induces significant local and regional changes in the ab initio model of chain E. Specifically, N501Y leads to substantial alterations in protein folding and notable changes in structural integrity. These modifications are more pronounced when compared to the wildtype structure, indicating that the N501Y mutation substantially impacts the RBD’s conformation. Such structural changes suggest that this mutation may have considerable implications for the RBD’s function, potentially affecting viral behavior and interactions with host cells.

In addition, our research, as demonstrated in Figure 10, further illustrates that even small local perturbations can significantly affect the overall structure of the spike protein’s RBD. Both L452R and N501Y mutations disrupt protein folding and secondary structures of the SARS-CoV-2 spike protein RBD, as evident from our results in Figures 10-12. Consistent with research findings, the L452R mutation, at a local level, introduces a positively charged residue that alters hydrophobic interactions and stability. On a global scale, it enhances spike protein stability, promotes viral fusion with host membranes, and strengthens ACE2 receptor binding [105]. Similarly, the N501Y mutation is known to form additional hydrogen bonds and π-π interactions with ACE2 locally while globally shifting the spike protein into an “open” prefusion conformation that facilitates receptor engagement. Collectively, these mutations increase ACE2-binding affinity, enhance viral infectivity and transmissibility, and contribute to immune evasion by reducing neutralizing antibody recognition.

Conclusively, our ab initio models offer a valuable structural framework for interpreting experimental data on these mutations. They also provide hypotheses for future investigations into their functional consequences, such as altered receptor binding or antibody recognition. This research contributes significantly to our understanding of how SARS-CoV-2 mutations affect the virus’ structure and function, potentially informing future strategies for treatment and prevention.

To gain further insights into the structural stability and dynamic behavior of both wildtype and mutated spike proteins, we conducted 3 sets of MD simulation studies at 50, 100, and 200 nanoseconds. Our results from MD simulations presented in Figure 13 provide a foundational understanding of short-, intermediate-, and long-term dynamics and structural changes for both wildtype and mutated spike proteins. All simulations offered valuable additional insights into conformational changes and stability patterns, demonstrating the high levels of instability of the mutated spike proteins when compared to the wildtype, thus providing a more robust independent confirmation of our graph-theoretic and ab initio model promising predictive analytic tools for complex networks and systems biology. These MD studies complemented our static structural analyses, offering a dynamic perspective on the effects of mutations on the spike protein’s behavior over time. Figure 13 displays the overlaid RMSD plots comparing the structural stability of wildtype (black) and mutated SARS-CoV-2 spike proteins (E484A: green; L452R: pink; K417N: lavender) across MD simulation timescales of 50 nanoseconds, 100 nanoseconds, and 200 nanoseconds. For each panel, mean RMSD values are indicated: at 50 ns, wildtype (0.1838, SEM 0.000381), E484A (0.2435, SEM 0.000727), L452R (0.3224, SEM 0.000984), K417N (0.2492, SEM 0.000624); at 100 ns, wildtype (0.2351, SEM 0.000495), E484A (0.3108, SEM 0.000896), L452R (0.4151, SEM 0.000957), K417N (0.3362, SEM 0.000897); at 200 ns, wildtype (0.1838, SEM 0.000381), E484A (0.2435, SEM 0.000727), L452R (0.3224, SEM 0.000984), K417N (0.2492, SEM 0.000624). The plot demonstrates the minimal fluctuation and high stability of the wildtype protein across all timescales, while mutants—most notably L452R—show significantly higher RMSD means, reflecting greater conformational variability and instability, especially at intermediate and longer durations.

To further validate our initial findings, we conducted extended MD simulations up to 100 nanoseconds and 200 ns, which revealed significant differences in structural stability for most mutant structures compared to the wildtype across these timescales, highlighting the mutation- and timescale-dependent destabilization of the spike protein structure. E484A, K417N, and L452R mutations consistently displayed higher RMSD values, indicating increased instability compared to the wildtype, with L452R demonstrating the highest instability among severe mutations. In contrast, the N440K mutation showed lower RMSD means, suggesting milder effects on protein stability. These findings indicate that mutations can substantially impact the structural dynamics of the spike protein, potentially affecting its function and interactions with host receptors. The observed changes in structural stability across different mutations correlate with clinical observations, suggesting a mechanistic link between altered protein dynamics and enhanced viral properties [105,106,109,110]. Specifically, these structural changes may contribute to the increased infectivity, immune evasion capabilities, and resistance to therapeutic antibodies observed in variants carrying these mutations. This alignment between MD simulations and clinical data underscores the importance of studying protein structural changes in understanding and predicting the behavior of SARS-CoV-2 variants [105,106,109,110].

The overlaid RMSD plot for the N440K (olive) and N501Y (indigo) variants compares their conformational stability with the wildtype spike protein across 50-nanosecond, 100-nanosecond, and 200-nanosecond simulations. For N440K, mean values were 0.2230 (SEM 0.000480) at 50 nanoseconds, 0.2123 (SEM 0.000862) at 100 nanoseconds (the lowest mean among all mutants at this timescale), and 0.2796 (SEM 0.000353) at 200 nanoseconds (SD 0.02494), with increasing mean and range at longer duration signifying greater fluctuation; at 200 nanoseconds, N440K displayed significant deviation from wildtype (Kolmogorov-Smirnov *P*=2.20×10^–16^). The N501Y mutation yielded mean RMSDs of 0.2279 (SEM 0.000559) at both 50 nanoseconds and 200 nanoseconds, and 0.2529 (SEM 0.000484) at 100 nanoseconds, indicating persistent but moderate structural alteration relative to wildtype. Statistical analyses confirmed significant conformational deviations for both mutations across timescales (*P*<2.2×10^–16^ at α=.05), except for N440K (*P*=1.43×10^–23^) and N501Y (*P*=1.18×10^–64^) at 100 nanoseconds, where deviations were less pronounced, as represented in Figures13  14.

To further validate our initial findings, we conducted extended MD simulations up to 100 nanoseconds and 200 nanoseconds, which revealed significant differences in structural stability for most mutant structures compared to the wildtype across these timescales, highlighting the mutation- and timescale-dependent destabilization of the spike protein structure. E484A, K417N, and L452R mutations consistently displayed higher RMSD values, indicating increased instability compared to the wildtype, with L452R demonstrating the highest instability among severe mutations. In contrast, the N440K mutation showed lower RMSD means, suggesting milder effects on protein stability. These findings indicate that mutations can substantially impact the structural dynamics of the spike protein, potentially affecting its function and interactions with host receptors. The observed changes in structural stability across different mutations correlate with clinical observations, suggesting a mechanistic link between altered protein dynamics and enhanced viral properties [105,106,109,110]. Specifically, these structural changes may contribute to the increased infectivity, immune evasion capabilities, and resistance to therapeutic antibodies observed in variants carrying these mutations. This alignment between MD simulations and clinical data underscores the importance of studying protein structural changes in understanding and predicting the behavior of SARS-CoV-2 variants [105,106,109,110].

### Comparison With Previous Work

A few studies have applied graph-theoretic models to investigate the effects of point mutations on protein structures and their associated disease phenotypes [84,85,107]. This research on SARS-CoV-2 spike protein mutations, particularly N501Y, L452R, E484A, and K147N, provides a detailed computational molecular analysis of the structural and functional alterations induced by these mutations. Specifically, it highlights the impact of mutations on the RBD of the spike protein, quantified using Euclidean distances and MD simulations. For instance, the N501Y and L452R mutations significantly disrupt protein folding, enhance ACE2 binding affinity, and contribute to immune evasion [21,28,29,41]. These findings align with previous studies like Knisley et al’s [113] graph-theoretic analysis of cystic fibrosis mutations in NBD1 of CFTR proteins, which quantified local and global structural changes caused by mutations. However, unlike previous studies that relied solely on graph-theoretic metrics to model structural perturbations in CFTR proteins [22,84,85,107-109], this research integrated MD simulations to compute RMSD values for mutated spike proteins in comparison to the wildtype. This approach reveals varying degrees of instability among the analyzed mutations, providing insights into mutation-induced conformational dynamics absent in earlier works. Similarly, while Kakraba and Knisley [60,85] focused on CFTR mutations in NBD2, and Netsey et al [61] examined point mutations like Glu6Val in sickle cell hemoglobin [85], this study uniquely addresses structural changes in SARS-CoV-2 spike protein RBD mutations. By combining quantitative approaches with molecular-level dynamics, this research offers additional strengths over previous methodologies that primarily used graph-theoretic models to analyze mutation effects [84,85,107]. Together, these complementary studies underscore the diverse applications of computational methods in unraveling mutation-driven phenomena across biological systems. While previous research has addressed distinct biological contexts, such as cystic fibrosis and SCD, this study emphasizes the importance of integrating multifaceted approaches, such as ab initio modeling and MD simulations with graph-theoretic modeling, to achieve a deeper understanding of mutation-induced structural and functional changes.

### Limitations and Future Directions

Despite these valuable insights from graph-theoretic analysis and MD simulations, certain limitations remain with this study. Specifically, our graph-based model did not fully capture the impact of mutations like N440K—associated with increased infectivity and immune evasion—as it may require more comprehensive molecular descriptors [107,108]. To address this concern, future research should aim to refine these models for better representation of such mutations. In addition, other mutations like S477N and T478K might be included in future studies. Also, future studies can use our graph-theoretic modeling approach to predict point mutations with potentially devastating consequences by analyzing changes in vertex weights and combinatorial descriptors in protein structure graphs, thereby providing a valuable tool for disease surveillance and early intervention strategies. Another limitation of this study is that our MD simulations focus solely on the isolated spike protein (chain E) of SARS-CoV-2, without considering its interaction with ACE2 or other ligands. As a result, we were unable to assess the direct impact of mutations on binding affinity or calculate binding energies, such as DeltaG, for the spike-ACE2 complex. Future studies could address this limitation by conducting molecular simulations of the spike protein bound to ACE2, which would provide deeper insights into how specific mutations affect their interaction and binding dynamics. More so, incorporating transmembrane domains and membrane anchoring in such models could further enhance the physiological relevance of the findings. We recognize that the appropriateness and impact of our chosen vertex-weighting scheme (ΔMd per degree) would benefit from systematic benchmarking against more established alternatives, such as hydrophobicity scores, residue centrality measures, and B-factor–based weights. While a comprehensive, side-by-side comparison of these metrics is beyond the scope of the present dataset and analysis, we acknowledge this as a limitation and a key opportunity for future work. Moving forward, we plan to undertake such comparative evaluations to more rigorously justify the selection of our weighting strategy. We also encourage others in the field to explore and refine these benchmarking efforts to better elucidate the strengths and limitations of alternative vertex-weighting methodologies within graph-theoretic modeling of protein mutation effects.

### Conclusions

This study provides a detailed computational analysis of key SARS-CoV-2 spike protein mutations, including N501Y, L452R, E484A, and K147N, and their structural and functional impacts. Using dendrogram clustering, Euclidean distance measurements, and MD simulations, the research highlights how these mutations disrupt protein stability and alter RBD. Mutations, such as N501Y and L452R, significantly enhance ACE2 binding affinity, viral transmissibility, and immune evasion, while even milder mutations like E484A and K147N contribute to structural perturbations and reduced antibody recognition. RMSD analysis revealed varying degrees of instability among mutated proteins, with L452R causing the greatest disruption. These findings align with clinical observations of increased infectivity and immune resistance associated with these mutations. While the study underscores the usability of computational models in understanding mutation-driven phenomena, it also highlights areas for future research, such as refining models to better capture the effects of additional mutations like S477N and T478K.

Overall, this research advances our understanding of SARS-CoV-2 evolution and provides critical insights for monitoring viral mutations and developing effective therapeutic strategies.

## Supplementary material

10.2196/73637 Multimedia Appendix 1 Structural analysis and molecular dynamics of SARS-CoV-2 spike receptor-binding domain mutations.

## Abbreviations

ACE2: angiotensin-converting enzyme 2
AD: Alzheimer disease
hACE2: human angiotensin-converting enzyme 2
I-TASSER: Iterative Threading Assembly Refinement
MD: molecular dynamics
OPLS-AA: Optimized Potentials for Liquid Simulations—All Atom
PDB: Protein Data Bank
RBD: receptor-binding domain
RMSD: root mean square deviation
SCD: sickle cell disease
SEM: standard error of the mean
SPC: simple point charge
SPC/E: simple point charge/extended
VOC: variants of concern
