# Correction: Structural and Functional Impacts of SARS-CoV-2 Spike Protein Mutations: Insights From Predictive Modeling and Analytics

**DOI:** 10.2196/89673

**Published:** 2025-12-29

**Authors:** Edem K Netsey, Samuel M Naandam, Joseph Asante Jnr, Kuukua E Abraham, Aayire C Yadem, Gabriel Owusu, Jeffrey G Shaffer, Sudesh K Srivastav, Seydou Doumbia, Ellis Owusu-Dabo, Chris E Morkle, Desmond Yemeh, Stephen Manortey, Ernest Yankson, Mamadou Sangare, Samuel Kakraba

**Affiliations:** 1 Department of Mathematics and Information Communication Technology, Dambai College of Education, Dambai, Ghana; 2 Department of Mathematics, School of Physical Sciences, University of Cape Coast, Cape Coast, Ghana; 3 Department of Geriatrics, School of Medicine, University of Arkansas for Medical Sciences, Little Rock, AR, United States; 4 Department of Mathematics, Memphis Shelby County Schools, Memphis, TN, United States; 5 Research and Development, CytoAstra LLC, Little Rock, AR, United States; 6 Office of Research, Celia Scott Weatherhead School of Public Health and Tropical Medicine at Tulane University, New Orleans, LA, United States; 7 Department of Biostatistics and Data Science, Celia Scott Weatherhead School of Public Health and Tropical Medicine at Tulane University, 1440 Canal Street, New Orleans, LA, 70112, United States, 1 5049882475; 8 Department of Public Health, Faculty of Medicine, Malaria Research and Training Center, University of Sciences, Techniques and Technologies of Bamako, Bamako, Mali; 9 School of Public Health, Kwame Nkrumah University of Science and Technology, Kumasi, Ghana; 10 Department of Mathematics, Suhum Senior High School, Suhum, Ghana; 11 Tulane Center for Aging, School of Medicine, Tulane University, New Orleans, LA, United States; 12 Department of Community Health, Ensign Global University, Kpong, Ghana; 13 Laboratory of Malaria and Vector Research (LMVR), National Institute of Allergy and Infectious Diseases (NIAID), Rockville, MD, United States

In “Structural and Functional Impacts of SARS-CoV-2 Spike Protein Mutations: Insights From Predictive Modeling and Analytics” [1], the authors noted four errors.

Author JAJ has now been marked as an equal contributor, as indicated by the asterisk added to their name.

Affiliation 1 has been changed from the following:

Department of Mathematics and Information Communication Technology, School of Physical Sciences, Dambai College of Education, Dambai, Ghana

The affiliation now reads:

Department of Mathematics and Information Communication Technology, Dambai College of Education, Dambai, Ghana

Affiliation 11 has been changed from the following:

Interdisciplinary Aging Studies, Tulane Center for Aging, School of Medicine, Tulane University, New Orleans, LA, United States

The affiliation now reads:

Tulane Center for Aging, School of Medicine, Tulane University, New Orleans, LA, United States

Affiliation 13 has been changed from the following:

Department of National Institute of Allergy and Infection Diseases (NIAID), 14 Laboratory of Malaria and Vector Research (LMVR), National Institute of Allergy and Infectious Diseases (NIAID), Rockville, MD, United States

The affiliation now reads:

Laboratory of Malaria and Vector Research (LMVR), National Institute of Allergy and Infectious Diseases (NIAID), Rockville, MD, United States

The corrections will appear in the online version of the paper on the JMIR Publications website, together with the publication of this correction notice. Because these were made after submission to PubMed, PubMed Central, and other full-text repositories, the corrected article has also been resubmitted to those repositories.
