# Supplementary material for: Structural and Functional Impacts of SARS-CoV-2 Spike Protein Mutations: Insights From Predictive Modeling and Analytics
Source: JMIR Bioinform Biotechnol. 2025 Dec 8;6:e73637. doi: 10.2196/73637 (PMC12685290; doi:10.2196/73637)
Supplement: Multimedia Appendix 1 [file bioinform-v6-e73637-s001.docx]

### Supplementary Files

**Supplementary Information: Structural Analysis and Molecular Dynamics of SARS-CoV-2 Spike RBD Mutations**

**Edem K. Netsey^1, †^, Samuel M. Naandam^2^, Joseph Asante^3^, Kuukua E. Abraham^4^, Aayire C. Yadem^5^,** **Gabriel Owusu^6^, Jeffrey G. Shaffer^7^, Sudesh K. Srivastav^7^** **Seydou Doumbia^8,9^, Ellis Owusu-Dabo^10^, Chris E. Morkle^11^, Desmond Yemeh^12^, Stephen Manortey^13^, Ernest Yankson^2^, Mamadou Sangare^14^ and Samuel Kakraba ^7,12, †,^ ***

1. Department of Mathematics and Information Communication Technology, Dambai College of Education, Dambai, 00233, Ghana; edem.netsey@stu.ucc.edu.gh (E.K.N.)
2. Department of Mathematics, University of Cape Coast, Cape Coast, 00233, Ghana; snaandam@ucc.edu.gh (S.M.N.); ernest.yankson@ucc.edu.gh (E.Y.)
3. Department of Geriatrics, University of Arkansas for Medical Sciences, Little Rock, AR 72205, USA; jasante@uams.edu (J.A.J.)
4. Department of Mathematics, Woodale High School, 5151 Scottsdale Ave, Memphis, TN 38118, USA; abrahamk1@scsk12.org (K.E.A.)
5. CytoAstra LLC, Little Rock, AR, USA; clement@cytoastra.com (A.C.Y.)
6. Office of Research Proposal Development, Tulane University Celia Scott Weatherhead School of Public Health and Tropical Medicine, Tulane University, New Orleans, LA 70112, USA; gowusu1@tulane.edu (G.O.)
7. Department of Biostatistics and Data Science, Tulane University Celia Scott Weatherhead School of Public Health and Tropical Medicine, Tulane University, New Orleans, LA 70112, USA; jshaffer@tulane.edu (J.G.S.); ssrivas@tulane.edu (S.K.S.); skakraba@tulane.edu (S.K.)
8. ‎Malaria Research and Training Center, University of Sciences, Techniques and Technologies of Bamako, Bamako, 00223 Mali; sdoumbi@gmail.com (S.D.)
9. University Clinical Research Center, University of Sciences, Techniques and Technologies of Bamako, Bamako, 0223, Mali
10. Department of Global and International School of Public Health, Kwame Nkrumah University of Science and Technology, Kumasi, 00233, Ghana; eowusu-dabo.chs@knust.edu.gh (E.O.D.)
11. Department of Mathematics, Suhum Senior High Technical School, 00233, Ghana; morklechris@gmail.com (C.E.M.)
12. Tulane Center for Aging, School of Medicine, Tulane University, 1430 Tulane Ave. 8513. New Orleans, LA 70112, USA; dyemeh@tulane.edu (D.Y.); skakraba@tulane.edu (S.K.)
13. Ensign Global University, Tema Akosombo Highway, Kpong(E/R), 00233, Ghana; steve.manortey@ensign.edu.gh (S.M.)
14. Laboratory of Malaria and Vector Research (LMVR), National Institute of Allergy and Infectious Diseases (NIAID), Rockville, Maryland, MD 20852, USA; mamadou.sangare@nih.gov (M.S.)

***** Correspondence: Samuel Kakraba, Ph.D.; [skakraba@tulane.edu](mailto:skakraba@tulane.edu);Tel.: +1-504-988-2475

^†^ These authors contributed equally to this work.

## Subdomain graphs of SARS-CoV-2 Spike RBD Chain E

After partitioning the sequence into subsequences as shown in Table 1, we employed a sophisticated multi-step graph-theoretic modeling approach. We generated *ab initio* models for each subsequence using I-TASSER[1], a cutting-edge protein structure prediction tool. The modeling process utilized a proximity threshold of 6 angstroms and determined endpoints based on each amino acid residue's center of mass. We then visualized these structural predictions using Cystoscape[2], resulting in 19 comprehensive subdomain graphs G1-G19. Each subdomain graph corresponds to a previously classified subsequence in Table 1, providing a detailed visual representation of the structural and interaction patterns within each RBD subdomain.

## Extended Molecular Dynamics Simulations of Wild-Type and Mutant SARS-CoV-2 Spike Proteins in Aqueous Solution Across 50 ns, 100 ns, and 200 ns Timescales

To further validate our findings, we conducted confirmatory molecular dynamics (MD) simulations extending over 100 nanoseconds, with additional analyses at 50 ns and 200 ns timescales. These simulations followed the same methodological approach as our initial MD studies, but with extended timeframes to provide a more comprehensive assessment of protein behavior. The primary objective was to determine whether the mutated proteins would exhibit differential conformational patterns compared to the wildtype over longer simulation periods. The extended simulations, including the 100 and 200 ns timeframe, were performed via WebGRO for Macromolecular Simulations[3]. We maintained consistent parameters with our initial simulations, including temperature, pressure, and solvent conditions. Trajectories were analyzed to compare the conformational dynamics of mutant and wildtype proteins, focusing on metrics such as root mean square deviation (RMSD) changes over time. This confirmatory test allowed us to observe long-term structural stability and conformational changes, providing a more robust validation of the differential behavior between mutated and wildtype spike proteins observed in our initial analyses. Figure 14 depicts the overlaid RMSD plots for 50 ns, 100 ns, and 200 ns simulations, as detailed in the legend for Figure 9.

**References**

1. Zhang, Y., *I-TASSER server for protein 3D structure prediction.* BMC Bioinformatics, 2008. **9**(1): p. 40.

2. Shannon, P., et al., *Cytoscape: a software environment for integrated models of biomolecular interaction networks.* Genome Res, 2003. **13**(11): p. 2498-504.

3. *WebGRO for Macromolecular Simulations* Available from: <https://simlab.uams.edu/>.
